# The Use of Mobile Apps in Adolescent Psychotherapy: Assessment of Psychotherapists’ Perspectives

**DOI:** 10.2196/65788

**Published:** 2025-04-08

**Authors:** Sarah Wüllner, Katharin Hermenau, Tobias Hecker, Michael Siniatchkin

**Affiliations:** 1 University Clinic of Child and Adolescent Psychiatry and Psychotherapy Medical School EWL, Protestant Hospital Bethel Bielefeld University Bielefeld Germany; 2 Institute for Interdisciplinary Conflict and Violence Research Bielefeld University Bielefeld Germany; 3 Department of Psychology Faculty of Psychology and Sports Science Bielefeld University Bielefeld Germany; 4 Department of Psychiatry Psychotherapy and Psychosomatics of Children and Adolescents University Hospital Rheinisch-Westfälische Technische Hochschule Aachen Aachen Germany

**Keywords:** mental health app, psychotherapy, adolescent, mHealth, youth, feasibility, implementation, app features, barriers, drivers

## Abstract

**Background:**

Therapy-accompanying mental health apps can play an important role in the psychotherapeutic treatment of adolescents. They can enhance adolescents’ engagement and autonomy, provide immediate support in critical situations, and positively influence the therapeutic working alliance. Nevertheless, mental health apps are rarely used by psychotherapists. Furthermore, due to the limited or nonexistent use of apps in psychotherapy, little is known about the actual barriers and drivers affecting their integration into psychotherapists’ daily routines. To better understand how mental health apps should be designed for practical use, it is essential to explore psychotherapists’ perspectives on key app features and characteristics, as well as the factors influencing their integration into clinical practice.

**Objective:**

This study aims to analyze which app features and characteristics are essential for psychotherapists to use a mobile app in psychotherapy with adolescents and to identify the key drivers and barriers influencing the integration of a psychotherapeutic app from the psychotherapists’ perspectives.

**Methods:**

We conducted 3 feasibility studies using Steps, a transdiagnostic, therapy-accompanying app for adolescents, across 3 different psychotherapeutic treatment contexts: inpatient treatment, treatment in psychiatric outpatient clinics, and outpatient treatment with psychotherapists in private practice. All studies followed a qualitative quasi-experimental design. Participants provided information on their age, occupation, years of work experience, media affinity, attitudes toward psychotherapeutic apps, perceived app quality and feasibility, and the implementation process of the therapy-accompanying app. Qualitative data were analyzed using deductive qualitative content analysis. A total of 40 mental health professionals participated across the 3 studies (study 1: n=18; study 2: n=13; study 3: n=9).

**Results:**

Study participation and app usage rates were low across all studies. Six core features for a transdiagnostic, therapy-accompanying app were identified: mood checks, library, reminders, goals and tasks, emergency kit, and questionnaires. Additionally, the integration of mental health apps into daily routines was influenced by various drivers and barriers. The most significant barriers included technological issues and practical constraints, such as limited time and resources. The most important driver was the perceived improvement in treatment quality.

**Conclusions:**

Overall, psychotherapists were generally open to using a therapy-accompanying mental health app. However, study participation and app usage remained low. As psychotherapists act as gatekeepers for patients’ use of mental health apps, their needs should be prioritized in the development and implementation of such apps.

**Trial Registration:**

German Clinical Trials Register DRKS00031258; https://drks.de/search/en/trial/DRKS00031258/details

## Introduction

### Background

There is a high risk of developing mental disorders during adolescence, which may persist into adulthood [1-3]. According to the World Health Organization (WHO), one in seven 10- to 19-year-olds experiences a mental disorder [4]. In addition, the rates of treatment nonresponse and therapy dropout are high among adolescents [5-7]. A promising way to address these challenges in psychotherapeutic care is the use of therapy-accompanying mental health apps (MHAs) [8-10]. At the latest, the pandemic has demonstrated that MHAs can play an important role in the psychotherapeutic treatment of adolescents [11-13] and are widely accepted by them [11,14,15].

Therapy-accompanying MHAs are used as an adjunct to psychotherapy [16]. They can offer specific manualized treatments or therapy tools that can be individually incorporated into psychotherapy [17,18]. The majority of available MHAs focus on specific disorders rather than a transdiagnostic therapy approach [12,17-19]. However, most MHAs have demonstrated the potential to provide transdiagnostic support beyond their primarily targeted disorder, as each contains fundamentally transdiagnostic features [18,20,21]. Additionally, to date, all therapy-accompanying apps have been designed for outpatient treatment [16,18]. To the best of our knowledge, no MHA has been specifically designed for inpatient treatment.

Therapy-accompanying MHAs offer several advantages in adolescent psychotherapy. They can enhance engagement [8-10] and promote greater autonomy [22,23]. Moreover, MHAs provide immediate support in critical situations, such as self-harm or acute suicidal ideation [16]. As a result, adolescents experience increased self-efficacy and improved therapeutic transfer to everyday life [8,16,17]. Furthermore, MHAs can have a beneficial influence on the therapeutic working alliance [8]. For psychotherapists, MHAs can facilitate documentation and monitoring of therapeutic progress, as well as communication with patients [24]. Another important aspect of integrating MHAs into psychotherapy is their effectiveness [24,25]. Previous research has shown that MHAs, in general, are effective in adolescent psychotherapy [18,26-35].

Although there are many reasons to use MHAs as an adjunct to psychotherapy with adolescents, they are rarely utilized by psychotherapists [24,36-41]. In 2019, only 9% of German health care professionals had tried an MHA in their psychotherapeutic practice [24]. The COVID-19 pandemic led to a slight increase in usage [37,39]; however, adoption remains low, with only 29% of German health care professionals reporting that they have used an app in their practice [39]. This raises the question: “Where does the discrepancy between the perceived benefits of MHAs and their limited use in psychotherapy originate?”

**Figure 1:**
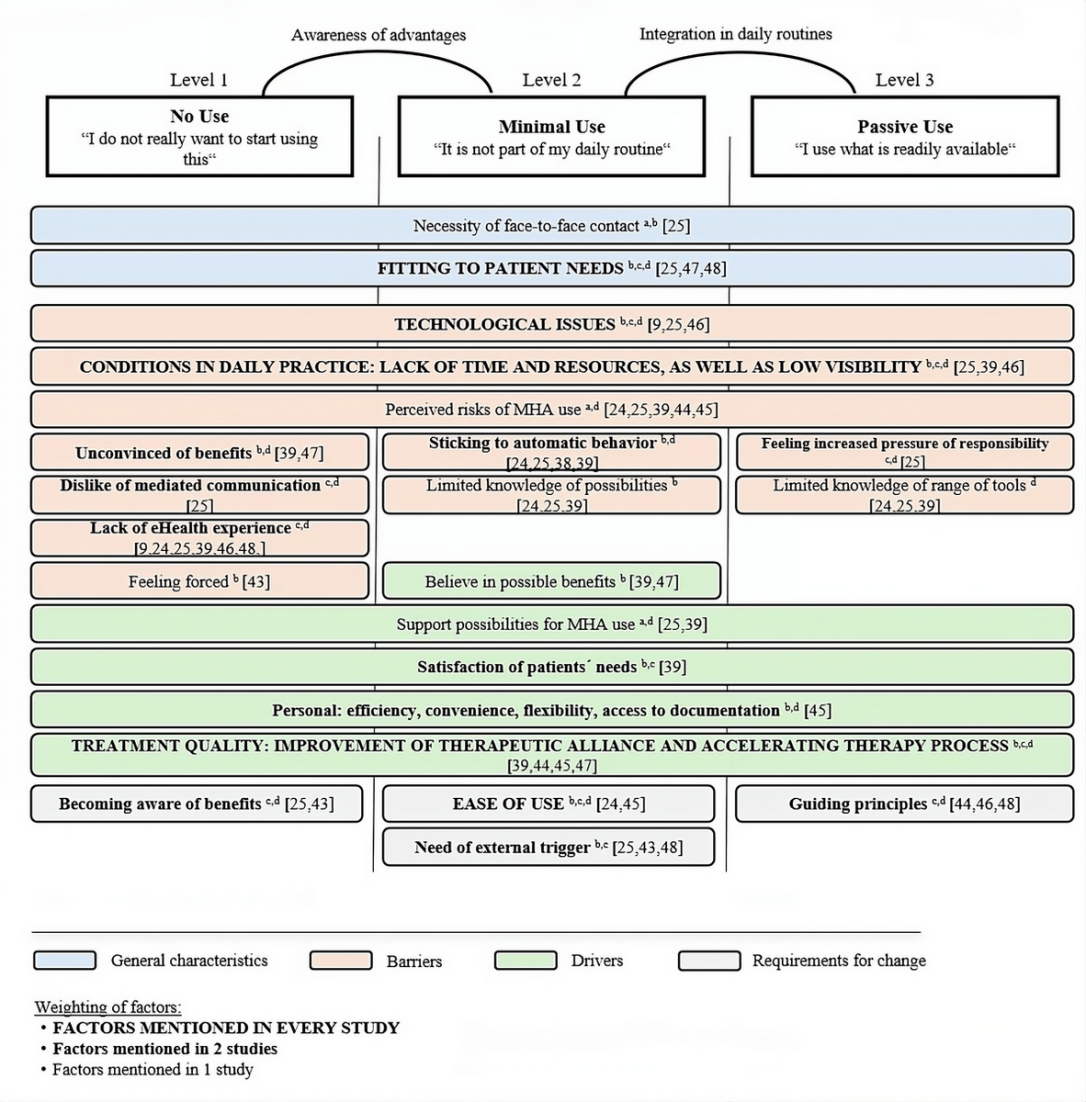
Levels of Adoption of e-Mental Health (LAMH) adapted for mental health app (MHA) use (aLAMH). The model is adapted from the LAMH model of Feijt et al. Factors that are marked with an "a" were added to the original model based on the current study results. Factors mentioned in study 1 are marked with "b"; factors mentioned in study 2 are marked with "c"; and factors mentioned in study 3 are marked with "d." The factors in the model were also mentioned in other studies as relevant association factors with psychotherapists' mental health app use.

To better understand this discrepancy, it is essential to identify the factors associated with the integration of MHAs into psychotherapists’ working routines. Feijt et al [42] developed a model to explain the factors influencing psychotherapists’ use of e-mental health: the Levels of Adoption of e-Mental Health (LAMH) model. This model categorizes influencing factors into different domains, including general characteristics, perceived drivers and barriers, and requirements for change. All factors are categorized according to different levels of e-mental health usage. The authors distinguish 5 levels of use, ranging from “no use” (level 1) to “innovative use” (level 5). According to previous research [24,39], psychotherapists generally fall between level 1 (no use) and level 3 (passive users) in their use of MHAs [42]. Consequently, our study focuses on the lower levels of adoption (levels 1-3), excluding level 4 (active use) and level 5 (innovative use). The selection of general characteristics, perceived drivers and barriers, and requirements for change mentioned in Feijt et al’s [42] is based on qualitative research results. Nevertheless, these factors have also been identified in other studies [9,24,25,39,43-48]. The LAMH model, adapted for this study and supplemented by our findings, is presented in Figure 1. It also includes corresponding references for each factor in the adapted LAMH model.

Like the development of the LAMH model, previous research on drivers and barriers has primarily focused on the theoretical perspectives of psychotherapists [9,24,44,45,47]. Furthermore, due to the limited or nonexistent use of apps in psychotherapy [37,39], little is known about the actual barriers and drivers influencing the integration of MHAs into psychotherapists’ working routines. Therefore, gaining a more practical understanding of these factors is essential.

In addition to questions about potential drivers and barriers to MHA integration, the design and features of MHAs may play a crucial role in psychotherapists’ use of these apps. What if psychotherapists do not use MHAs because the available apps do not sufficiently meet the needs of adolescent psychotherapeutic treatments? To better understand how MHAs should be designed for actual use in psychotherapists’ working routines, it is essential to explore their perspectives on the key features and characteristics of therapy-accompanying MHAs.

### Objectives

This study aims is to investigate how a therapy-accompanying MHA should be designed for successful integration into adolescent psychotherapy across different psychotherapeutic contexts. To assess psychotherapists’ perspectives in a more practical way, we conducted a needs evaluation within the initial feasibility pilot testing of a transdiagnostic, therapy-accompanying app for adolescents in 3 different treatment settings: inpatient treatment, psychiatric outpatient clinics, and outpatient treatment with private practice psychotherapists. We analyzed the essential app features and characteristics required for psychotherapists to use an MHA in adolescent psychotherapy, as well as the key drivers and barriers influencing its integration from the psychotherapists’ perspectives.

## Methods

### Overview

**Figure 2:**
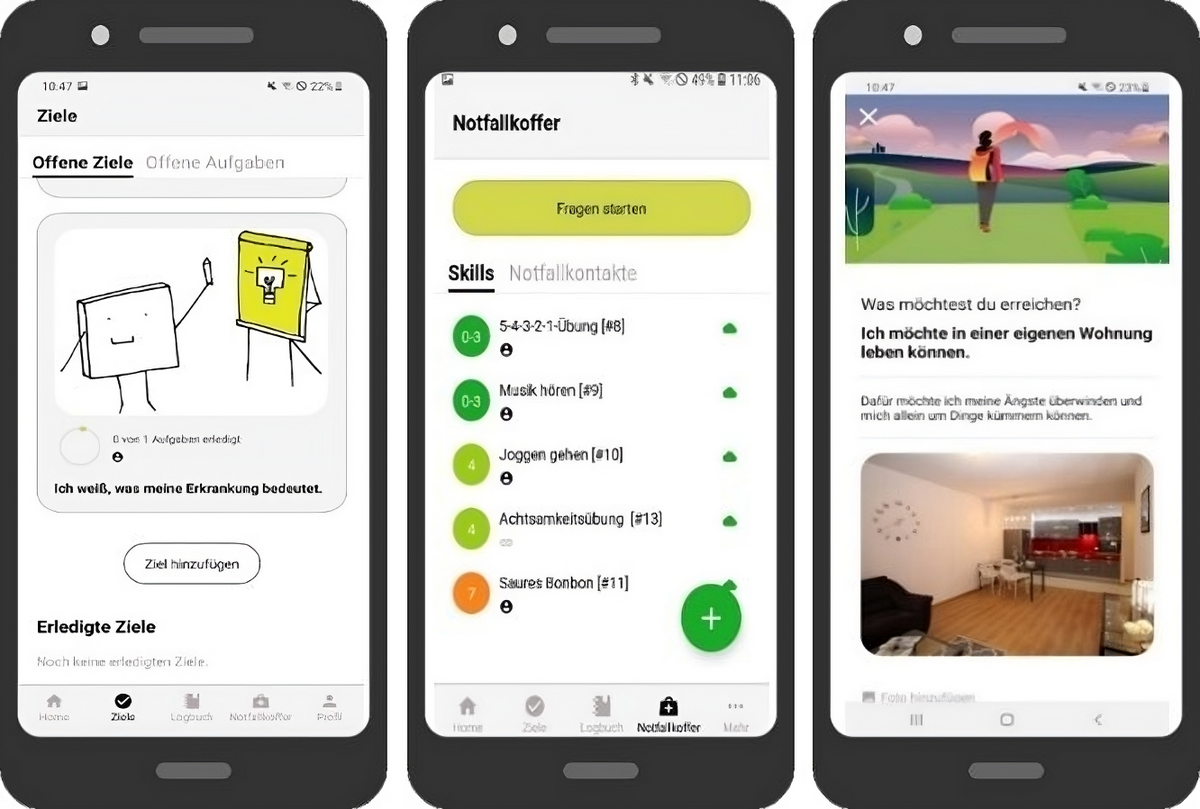
Screenshots of different app features of the patient version of the app Steps: goals and tasks (left), emergency kit (center), and the main therapy goal (right).

**Figure 3:**
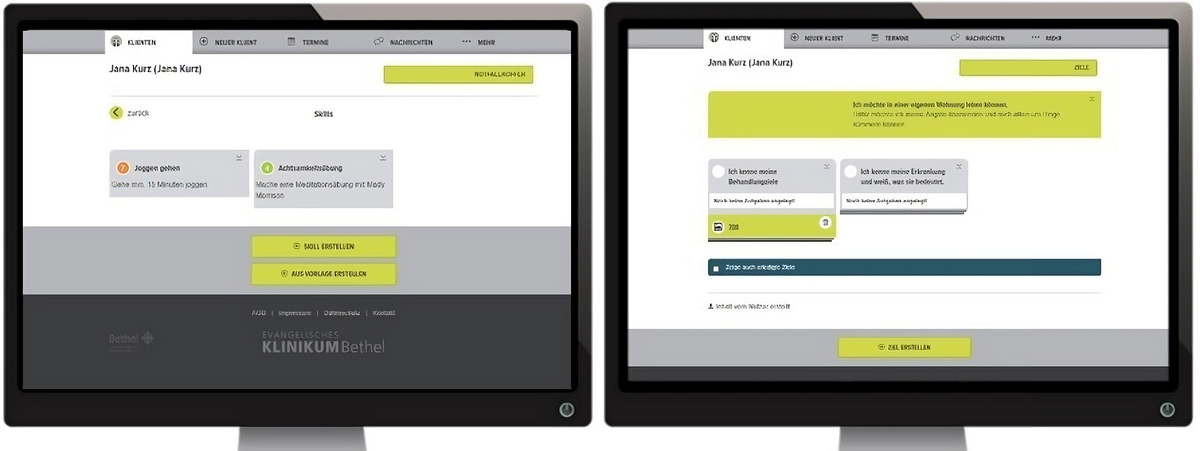
Displays of different app features of the web-based psychotherapist version of the app Steps: list of skills in the emergency kit (left) and goals and tasks (right).

Three feasibility studies were conducted in different settings using the transdiagnostic, therapy-accompanying app *Steps*. The app was designed as an adjunct to regular psychotherapeutic treatment for adolescents. It was developed through a participatory process by Circumradius GmbH and the Protestant Hospital Bethel in Bielefeld. Steps offered interconnected versions for both patients and their psychotherapists. While patients used the Steps app on their own Android-based smartphones, psychotherapists accessed a web-based version (see Figures 2 and 3). The app included various transdiagnostic features (Table 1). For implementation, psychotherapists were not required to follow specific usage rules or treatment manuals; they could independently decide to what extent they wanted to integrate the app into their psychotherapy sessions. All studies followed a qualitative quasi-experimental study design. In all studies, data were collected from both psychotherapists and patients. However, due to the objectives of this article and the comprehensiveness of the collected data, patient data were not included in the current analyses. Additionally, only methods and results relevant to this analysis are reported in this article.

**Table 1:** Overview of the provided app features in the therapy-accompanying and transdiagnostic app Steps for adolescent psychotherapy.

App feature	Description
Mood checks	Psychotherapists can customize mood checks for patients (type and number of items, response format, frequency of queries). Patients are prompted and reminded by the app to complete the mood checks. In the psychotherapist version, a graphical overview of the mood checks is available.
Main therapy goal	Patients can record what they want to achieve through their psychotherapy. Photos can be uploaded to visualize patients’ therapy motivation.
Intermediate therapy goals	Intermediate therapy goals can be set to structure or visualize therapy progress. Patients can complete individual tasks to achieve intermediate goals.
Tasks	Psychotherapists can set tasks for their patients, for example, to complete between therapy sessions. When patients have completed a task, they receive an automated reflection query. Therapists can directly access the information entered and easily refer to the situation-related information in the next therapy session.
Logbook	Mood checks done by patients are saved in the logbook. Patients can also create free logbook entries to record important events, or to document the therapy progress.
Questionnaire manager	Psychotherapists can set up questionnaires for their patients to complete directly in the app. Questionnaires can be customized by psychotherapists (type and number of items, response format, and frequency of queries). Answers to questionnaires can be downloaded directly in the psychotherapist version.
Emergency kit	Skills and emergency contacts can be saved in the emergency kit. Skills are sorted according to stress levels from 1 (low stress) to 10 (maximum stress). Photos, home page links, or address details can be added to skill entries. Optional: activation of queries for analysis of undesirable behavior (eg, nonsuicidal self-injuries).

### Design and Procedure

The first study examined the feasibility of using Steps in inpatient treatment for adolescents. Adolescents admitted as inpatients who consented to participate in the study received a clinic-owned smartphone for their treatment. This smartphone included the Steps app along with other treatment-relevant apps and was available for use throughout their entire treatment period. The entire staff of the adolescent ward was trained in app usage and instructed to integrate Steps into the treatment process. All employees were invited to participate in the study on a voluntary basis. Psychotherapists completed a baseline survey at the start of their participation and a follow-up questionnaire at the end of the data collection period. For other employees, all questions were consolidated into a single survey conducted at the end of the project. Additionally, 4 employees participated in qualitative interviews assessing the app’s quality and the implementation process of Steps.

The second study was conducted in psychiatric outpatient clinics. All staff members from the participating clinics were invited to take part. Based on the participation challenges encountered in the first study, psychotherapists were offered 2 options for participation: (1) actively using the app in treatment with adolescents and providing feedback on their experiences, or (2) providing feedback on Steps after a brief introduction without directly using the program. Each participant received individual app training to familiarize themselves with the app and its application in therapy. If psychotherapists incorporated the app into their treatment, participating patients were required to install Steps on their own smartphones. If needed, adolescents could borrow a clinic-owned smartphone for study participation. Data were collected from participating employees at the start of their study involvement and again at the end of the data collection period using the open-source online survey tool LimeSurvey (LimeSurvey Team) [49]. The study was preregistered in the German Clinical Trials Register (DRKS00031258).

The third study was conducted with psychotherapists in private practice. Interested psychotherapists were recruited through the Association of Child and Adolescent Psychotherapists of East Westphalia-Lippe. The study procedure was consistent with that of the second study, with one difference: data for the second assessment were collected through qualitative interviews.

### Ethical Considerations

All 3 studies received positive ethical approvals. Studies 1 and 2 were approved by the Ethics Committee of the Medical Association of Westphalia-Lippe (approval numbers 2020-878-f-S and 2022-528-f-S, respectively). Additionally, study 2 was approved by the Ethics Committee II of the University of Heidelberg (approval number 2022-643) and the Ethics Commission of the Faculty of Medicine at the University of Cologne (approval number 23-1026_1). Study 3 was approved by the Ethics Committee of Bielefeld University (approval number EUB-2023-050). In all 3 studies, participants received detailed study information sheets outlining the study procedures, research aims, and data protection guidelines. Participation was voluntary and confidential. Psychotherapists signed informed consent before their participation. To ensure data security, all participant data were collected in a pseudonymized manner. Personal data (eg, contact details or audiotapes) were stored separately from the pseudonymized data. Audiotapes of the qualitative interviews were transcribed. Once data analysis is complete, the audiotapes will be destroyed, and the data will be anonymized. Participants did not receive any compensation. This study followed the COREQ (Consolidated Criteria for Reporting Qualitative Research) checklist for study conceptualization, data analysis, and reporting.

### Participants

A total of 40 mental health professionals participated in the 3 studies. Sample characteristics varied based on the treatment context. In study 1, participants included 18 employees from a psychiatric ward for adolescents at a German clinic specializing in child and adolescent psychiatry and psychotherapy. In study 2, 13 employees from 3 different psychiatric outpatient clinics in Germany took part. In study 3, 9 psychotherapists in private practice who treated adolescents aged 12-18 years were included. Detailed sample characteristics are presented in Table 2.

**Table 2:** Overview of the sample characteristics presented separately for each study context.

Sample characteristics	Study 1: Inpatient treatment (n=18 participants)	Study 2: Psychiatric outpatient clinics (n=13 participants)	Study 3: Psychotherapy in private practices (n=9 participants)
Included occupational groups	Psychotherapists (n=3) Specialist therapists (eg, music therapists, n=3) Nursing professionals (n=11) Youth volunteer (n=1)	Psychotherapists (n=9) Psychologist without further training (n=1) Assistant doctors (n=3)	Psychotherapists (n=9)
Age (years), mean (SD); range	32.50 (9.59); 22-62	35.15 (9.05); 27-61	54.44 (13.79); 33-72
Work experience (years), mean (SD); range	8.33 (9.46); 0.60-40.50	6.08 (5.12); 2.33-16.42	23.44 (14.34); 6-40

### Measures

#### Survey Methodology and Questionnaire Design

In all studies, surveys were conducted using self-administered questionnaires, which included both quantitative and qualitative questions. Participants provided information on their age, occupation, years of work experience, media affinity, attitudes toward psychotherapeutic apps, perceived app quality and feasibility, and the implementation process of Steps. Most questions were purpose-built. Because of variations in study contexts and insights gained from previous studies, some questions and questionnaires differed between the studies. Detailed differences in the surveys are presented in Multimedia Appendix 1. In the inpatient treatment context (study 1), psychotherapists received different questionnaires than other employees to account for the distinct responsibilities and working areas of each occupational group.

#### Media Affinity and Attitudes Toward Psychotherapeutic Apps

In study 1, media affinity was assessed using the Affinity for Technology (TA-EG) questionnaire [50]. The TA-EG consists of 19 items across 4 subscales: enthusiasm for technology, competence in using technology, perceived positive consequences of technology, and perceived negative consequences of technology. Participants rated each item on a 5-point Likert scale from 1 (does not apply at all) to 5 (fully applies). Subscale scores were calculated as the mean of the associated items, ranging from 1 to 5 points. The internal consistency values for the TA-EG questionnaire are presented in Table 3. Based on the experiences from study 1 and the differing working conditions in outpatient therapy settings, media affinity was assessed differently in studies 2 and 3. Participants answered self-created questions about the technical equipment available at their workplace, their use of media and smartphone apps, and the integration of media into their psychotherapies. Additionally, in study 3, participants completed the Affinity for Technology Interaction (ATI) questionnaire [51], a unidimensional measurement instrument consisting of 9 items. Participants rated each item on a 5-point Likert scale from 1 (not true at all) to 5 (completely true). The overall ATI score was calculated as the mean value of all items, ranging from 1 to 5. The internal consistency of the ATI is presented in Table 3.

**Table 3:** Means, SDs, ranges, and reliability coefficients of the questionnaires on media affinity, attitudes toward e-mental health, and app quality from psychotherapists’ perspectives. The data are presented separately for each study context.

Questionnaire				n	Mean (SD)	Range	Actual scale range	Internal consistencies (Cronbach α)
**Study 1**								
	**TA-EG^a^**							
			Enthusiasm	18	2.93 (0.77)	1.60-4.40	1-5	0.90
			Competencies	18	3.42 (0.62)	2.50-4.75	1-5	0.63
			Positive consequences	18	4.01 (0.37)	3.40-4.80	1-5	0.57
			Negative consequences	18	3.02 (0.63)	1.80-4.20	1-5	0.83
**Study 2**								
	**uMARS^b^**							
			Overall	10	3.75 (0.60)	2.77-4.52	1-5	0.87-0.90^c^
			Engagement	11	3.76 (0.69)	2.70-4.80	1-5	0.76-0.82^c^
			Functionality	10	3.77 (0.85)	2.38-5.00	1-5	0.70-0.88^c^
			Aesthetics	10	3.76 (0.59)	3.00-4.58	1-5	0.37-0.47^c^
**Study 3**								
		ATI^d^		9	2.98 (0.99)	1.56-4.67	1-5	0.92
		**MTPS^e^**						
			Potential to augment psychotherapy	9	3.56 (0.46)	3.00-4.25	1-5	0.42
			Perceived risks	9	2.97 (0.85)	2.00-4.75	1-5	0.78
		**uMARS**						
			Overall	5	3.52 (0.56)	2.55-3.87	1-5	0.66-0.82^c^
			Engagement	9	3.79 (0.66)	3.00-4.86	1-5	0.50-0.82^c^
			Functionality	5	3.54 (0.90)	2.14-4.45	1-5	0.57-0.60^c^
			Aesthetics	9	3.58 (0.86)	2.30-5.00	1-5	0.58-0.76^c^

^a^TA-EG: Affinity for Technology [50].

^b^uMARS: user version of the Mobile Application Rating Scale [52].

^c^Internal consistencies of the app *Steps* (summarized for the app version and the web version of the app).

^d^ATI: Affinity for Technology Interaction [51].

^e^MTPS: Psychotherapists’ Attitudes Toward Using Modern Technologies in Psychotherapy and Counselling Scale [53].

Given the specific study context of inpatient treatment (study 1), attitudes toward psychotherapeutic apps were assessed using 12 self-created questions based on the Unified Theory of Acceptance and Usability of Technology (UTAUT) [54] and an adapted UTAUT questionnaire by Hennemann et al [14]. Participants responded to the items on a 5-point Likert scale, ranging from 1 (strongly disagree) to 5 (strongly agree). In study 2, the self-created questions were adapted to the outpatient study context. As a result, attitudes toward psychotherapeutic apps were measured using a 10-item questionnaire. In study 3, attitudes toward psychotherapeutic apps were measured using the Psychotherapists’ Attitudes Toward Using Modern Technologies in Psychotherapy and Counselling Scale (MTPS) [52]. The MTPS is a multidimensional self-report questionnaire with 16 items across 4 subscales: *potential to augment psychotherapy, psychoeducational value, perceived risks,* and *perceived relevance.* We included 2 subscales—*potential to augment psychotherapy* (eg, “Modern technologies can speed up the therapeutic process”) and *perceived risks* (eg, “A stricter regulation of the content available via modern technologies in relation to psychotherapy and psychopathology should be enforced”). Both scales are considered central factors in practitioners’ attitudes toward technology [52,55]. Psychotherapists rated their agreement with the statements on a 5-point Likert scale, ranging from 1 (strongly disagree) to 5 (strongly agree). Subscale scores were calculated as the mean values of the 4 associated items in each subscale, with scores ranging from 1 to 5. For this study, the MTPS was translated into German. The quality of the translation was ensured through multiple forward and backward translations. The internal consistencies of the subscales are presented in Table 3.

#### Perceived App Quality and Feasibility

As study 1 was the first evaluation study on Steps, perceived app quality and feasibility were assessed using purpose-built open questions, informed by previous evaluation studies [14,56-58]. The questions focused on app functionality, design, available features, and aspects of the implementation process (eg, “Steps makes my therapeutic work easier” or “The use of Steps fits well into everyday life on the ward”). To obtain a more distinct and structured assessment of perceived app quality and feasibility, 4 subscales of the user version of the Mobile Application Rating Scale (uMARS) [53] were used in studies 2 and 3: engagement, functionality, aesthetics, and subjective app quality. The uMARS is a self-report questionnaire that was translated into German for this study, with accuracy ensured through blind back-translation. Participants rated 17 items on a 5-point Likert scale (1=totally disagree and 5=fully agree). They evaluated both the patient version of the app and the web-based psychotherapist version of Steps. Subscale scores for *engagement*, *functionality*, and *aesthetics* were calculated as the mean value of the associated items for each subscale, ranging from 1 to 5. Five additional questions addressed *subjective app quality* (eg, “What is your overall [star] rating of the app?”). Overall app quality scores for both the app version and the web version were determined by the mean value of all items. The internal consistencies are presented in Table 3. In studies 2 and 3, the feasibility of Steps was assessed using purpose-built questions based on those used in study 1 but adapted to the contexts of psychiatric outpatient clinics and private practice psychotherapists.

### Data Analysis

Qualitative interviews from studies 1 and 3 were transcribed. All qualitative data, including interview transcripts and free-text responses from the surveys, were analyzed using deductive qualitative content analysis [59]. An initial set of categories was developed based on the uMARS and LAMH model before data analysis began. In the first step, 1 researcher coded the qualitative data. Following the deductive qualitative content analysis process described by Cho and Lee [59], data that did not fit into the predetermined categories were assigned to newly created categories. The final set of categories for the content analysis is presented in Multimedia Appendix 2. All qualitative data were recoded using the complete set of categories. Following Mayring [60], a second rater coded a subset of the qualitative data (2 qualitative interviews) to ensure coding clarity. Any discrepancies between the 2 raters were thoroughly discussed. The frequency of each category mentioned by participants, as well as the number of participants who reported each category, was assessed. Categories were ranked and reported based on their occurrence. For this publication, participant quotes from the study were translated into English. Descriptive quantitative data were analyzed using SPSS version 29.0.0.0 (IBM Corp.).

## Results

### Study Participation and Attitudes Toward MHAs

Study participation was low across all studies (Table 2). Despite extensive recruitment efforts, only a few psychotherapists chose to test the transdiagnostic, therapy-accompanying app Steps as part of the studies. Additionally, patient participation was low, and dropout rates were high. Detailed information on study participation and dropout rates is provided in Multimedia Appendix 3. In all studies, mental health professionals reported moderate media affinity and mixed attitudes toward MHAs. In studies 2 (psychiatric outpatient clinics) and 3 (psychotherapists in private practice), most psychotherapists had never recommended MHAs to patients before (study 2: 9/12 participants; study 3: 8/9 participants). Frequencies of the self-created questions from studies 2 and 3 regarding the technical equipment available at work, the use of media and smartphone apps, and the integration of media into psychotherapies are provided in Multimedia Appendix 3. Descriptive statistics for the standardized questionnaires used in the studies are presented in Table 3.

### Perceived App Quality and Feasibility of Steps

For inpatient treatment of adolescents, about half of the participants in study 1 rated the joy of use neutrally (n=10; fun to use: n=4, not fun to use: n=2) but found Steps helpful for patients (n=9; undecided: n=5, unhelpful: n=3). However, the app was rarely used for psychotherapeutic treatment. Almost all participants agreed that the app did not fit the inpatient context. Eight participants suggested that Steps might be more beneficial for outpatient treatments. Correspondingly, 1 participant mentioned using the app for follow-up care after patient discharge from the clinic.

I could imagine, [Steps] perhaps as a transitional app [...] to somehow bridge the time before therapy or as aftercare after therapy. [...]

WI2

By contrast, for outpatient treatment of adolescents, the majority of participants expressed interest in continuing to use the app for some patients after the project ended (study 2—yes, n=9; no, n=3; and missing, n=1; and study 3—yes, n=7 and no, n=2). Additionally, most psychotherapists indicated a willingness to recommend Steps to some colleagues (study 2—few, n=1; some, n=5; many, n=3; everyone, n=3; and missing, n=1; and study 3—few, n=1; some, n=5; many, n=2; and missing, n=1). However, while 5 psychotherapists in private practice were willing to pay for app use (no, n=4), only 1 participant from the psychiatric outpatient clinics expressed willingness to pay (no, n=10). Furthermore, Steps received an average star rating of 3.55 (SD 0.65; range 2.00-4.00; n=11) in study 2 and 3.19 (SD 0.53; range 2.50-4.00; n=8) in study 3. These ratings were comparable to those of other MHAs [21]. Descriptive statistics for the uMARS are presented in Table 3.

### Essential App Features

In the inpatient setting, the most frequently mentioned feature was setting therapy goals and tasks (n=4). While psychotherapists rated this feature as unhelpful and disliked the emphasis it placed on goals and tasks in the therapy process, nursing professionals and specialist therapists found it interesting to use.

I liked the visualization of the therapy process and successes [through the goals and tasks feature

W18

The second most frequently mentioned feature in the inpatient setting was the mood check (n=3). Additionally, setting reminders was identified as an important feature (n=4), with participants expressing a desire for greater flexibility.

It should be possible to set a reminder as flexible as in calendar apps [the current app version provided periods that were not detailed enough]

W18

The results from the outpatient therapy settings presented a different perspective. Employees of psychiatric outpatient clinics identified mood checks (mentioned 8 times by 5) and the emergency kit (mentioned 5 times by 5) as the most helpful features. Additionally, the questionnaire manager was considered useful for diagnostics if templates were available (mentioned 4 times by 4). However, participants were unwilling to spend time digitizing their own questionnaires.

[Providing questionnaires was] far too laborious to enter them yourself.

C14

The availability of templates was also highlighted for other app features. Three participants from psychiatric outpatient clinics suggested a library feature to provide psychoeducational materials, tests, skills, or templates for mood checks. Additionally, psychotherapists in private practices identified mood checks (n=8), the library (n=8), and the questionnaire manager as the most helpful features of a therapy-accompanying app. Additionally, psychotherapists in private practices highlighted the option to digitize therapy materials (n=8) and facilitate communication with patients through an app as particularly helpful. However, the communication feature was also perceived as challenging. Three psychotherapists expressed concerns about legal provisions and responsibilities, particularly in cases where patients might communicate indicators of suicidality. Furthermore, a chat feature was seen as potentially leading to an “increased workload that is not practicable” (P1) for psychotherapists. Similarly, feedback on reminders was mixed. While the majority of psychotherapists found it a helpful feature (n=6), 1 psychotherapist noted that it could potentially contradict therapeutic processes:

I somehow also expect patients to remember appointments and perhaps manage this well via a calendar or whatever, and otherwise it’s actually a good indication of motivation for therapy for me.

P2

**Figure 4:**
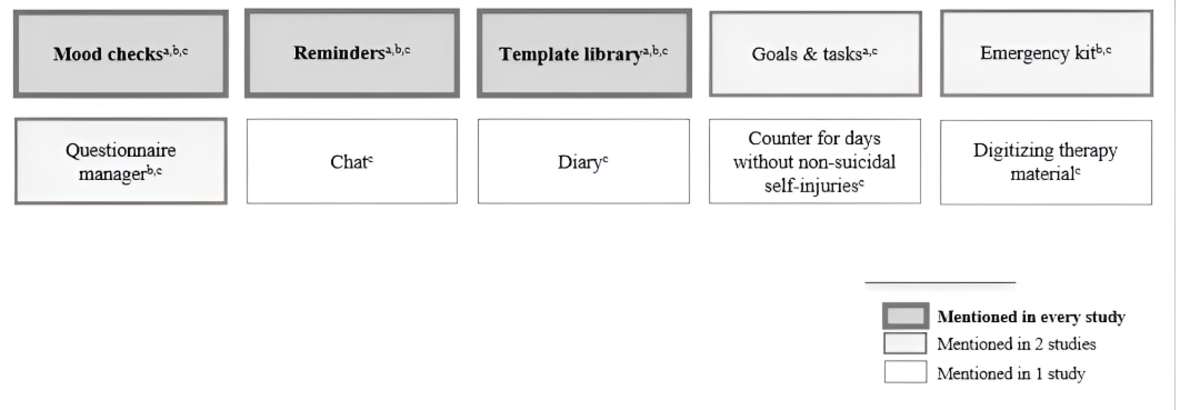
Essential app features for a transdiagnostic, therapy-accompanying app mentioned in the different studies. Features mentioned in study 1 are marked with "a"; features mentioned in study 2 are marked with "b"; and features mentioned in study 3 are marked with "c.".

Other app features suggested by psychotherapists in private practices included a tool for tracking days without nonsuicidal self-injury, the provision of relaxation exercises, and support for organizational therapy tasks, such as sending documents or completing necessary forms and questionnaires. An overview of the key app features mentioned across the 3 studies is presented in Figure 4.

### Essential App Characteristics

Across all study contexts, ease of use and customizability were identified as crucial app characteristics. Ease of use was mentioned by several participants in each study (study 1, n=4; study 2, n=6; and study 3, n=9). Half of the interviewed participants from the inpatient treatment setting emphasized the importance of customizability (study 2, n=4 and study 3, n=8). However, some participants from study 1 highlighted the benefits of preinstalled therapy tools and materials, such as activity lists, meditation and relaxation exercises, and mindfulness practices. Psychotherapists in private practices particularly valued the ability to adapt the app to different psychotherapeutic approaches and individual workflows, considering it a key strength of Steps.

also open to different psychotherapeutic approaches...to offer psychotherapists a tool...that...yes, incorporates modern technologies into the treatment and makes it usable for both psychotherapists and patients at the same time, so I think that’s an advantage of Steps.

P6

However, the ability to customize the app individually was also identified as a potential barrier by psychotherapists in private practices. They noted that digitizing materials and preparing personalized app usage require additional time. In line with this concern, 1 participant from the psychiatric outpatient clinics suggested implementing an easy copy function to facilitate the creation of custom app content (C1).

In addition, accessibility was highlighted as another crucial app characteristic. The absence of an iOS version was cited as the primary reason for not using Steps by 3 practitioners from psychiatric outpatient clinics and 3 psychotherapists in private practices. However, 1 participant from the psychiatric outpatient clinics and 8 out of 9 psychotherapists in private practices positively emphasized the benefit of both psychotherapists and patients having individual yet interrelated accounts within Steps.

I also think it’s good that there’s a psychotherapist app that I can use to control things to a certain extent. I think it’s good to be able to provide the patient with materials. Yes, I think so, for now.

P2

Regarding a therapy-accompanying app for inpatient treatment, all participants in the qualitative interviews emphasized that individual access to each professional role is essential (n=4).

Yes, and then I might have had more of a connection to it [with my access]. So, because I only had access via the adolescents, it was forgotten a bit quicker

WI3

The design was another crucial factor, with 5 participants from the inpatient therapy setting directly mentioning its importance. Additionally, 3 out of 4 interviewed participants from study 1 emphasized that design serves as a motivator for usage. In the outpatient therapy settings, design was also highlighted as significant. Two practitioners from the psychiatric outpatient clinics reported feeling less motivated to use Steps due to the unattractive design of the psychotherapist version. Feedback on the patient version of the app was mixed. On the one hand, the “[...]individual design and yet reduced to the essentials[...]” (C17) was mentioned positively. On the other hand, C18 suggested that the “layout could be more colorful” to motivate adolescents to use the app. In the sample of psychotherapists in private practices, 3 psychotherapists suggested a more playful design, such as a customizable avatar or a reward system, to make the app more appealing to adolescents. Additionally, a participant proposed incorporating motivational features into the psychotherapist version to enhance engagement.

Maybe there’s a nice seductiveness, so to speak. That a psychotherapist who doesn’t like looking at smartphones likes to look at them, [...].

P3

### Aspects for the Integration of MHAs Into Psychotherapists’ Daily Routines

In all studies, one of the most common barriers was *conditions in daily practice* (study 1: mentioned 25 times by 12 in the surveys and several times by 4; study 2: mentioned by 1; and study 3: mentioned 23 times by 8). In the inpatient therapy setting, other common barriers included *sticking to automatic behavior* (mentioned 9 times by 6) in the surveys, as well as being *unconvinced of benefits* (mentioned 14 times by 3) and *sticking to automatic behavior* (mentioned 7 times by 3) in the qualitative interviews. Regarding the barrier “sticking to automatic behavior”, WI1 of the inpatient therapy setting said:

How do I get what I have there now into this app, where I intuitively know how to proceed [without an app]? I didn’t have time for that, and the adolescents didn’t even think about it.

In line with the barriers mentioned in the inpatient therapy setting, the barrier “technical issues” was also reported by 2 practitioners in the psychiatric outpatient clinics, while the barrier “unconvinced of benefits” was mentioned 16 times by 7 psychotherapists in private practice. Additionally, psychotherapists in private practice also identified “perceived risks” as one of the most common barriers to app usage (mentioned 12 times by 6). In the psychiatric outpatient clinics, “technical issues” was likewise mentioned as one of the most common barriers by 2 practitioners.

The most common drivers varied across study contexts. In the inpatient therapy setting, the most frequently mentioned driver in both surveys and interviews was *approving of treatment quality*. Other drivers mentioned once included *satisfaction of client’s needs,* *personal efficiency,* and *belief in possible benefits.* By contrast, participants from the psychiatric outpatient clinics identified the potential to digitize aspects of psychotherapy as a driver for app use and an indicator of its suitability for client needs.

[...]because paper pencil questionnaires/mood checks etc. can be replaced. Young people are much more interested in this.

C5

Psychotherapists in private practices cited *personal efficiency* (15 times by 7), *approving of treatment quality* (14 times by 7), and *support offers for app use* (3 times by 3) as the 3 most common drivers. Regarding the general characteristics of app usage, all participants in the qualitative interviews of study 1 (n=4) and 8 out of 9 psychotherapists in private practices highlighted the app’s fit to client needs as a crucial factor for its use. Additionally, 2 psychotherapists in private practices emphasized the necessity of face-to-face contact during the relationship-building phase at the beginning of therapy as a general characteristic. Regarding the requirements for change, all interviewed participants in the inpatient therapy setting mentioned the need for external triggers to integrate an app into their daily work routines.

[It needs someone who] can consistently accompany such a project and also pick up [everyone of] the colleagues.

WI1

An overview of all mentioned drivers, barriers, general characteristics, and requirements for change in all 3 studies is presented in Figure 1.

## Discussion

### Principal Findings

Psychotherapists play an important role in integrating MHAs into adolescent psychotherapies [38,61]. However, previous research has indicated that apps are rarely used in psychotherapeutic treatments, even though mental health professionals are generally interested in MHAs [36-41]. In line with this, participating psychotherapists reported positive attitudes toward MHAs, but app usage rates were low across all study contexts. Furthermore, the results of the 3 studies indicated that psychotherapists had little to no experience using MHAs for psychotherapeutic treatment. To highlight the discrepancy between positive attitudes toward MHAs and their low level of use in psychotherapeutic treatment, we assessed, on the one hand, psychotherapists’ needs for a therapy-accompanying MHA and, on the other hand, potential factors influencing the integration of MHAs into their daily working routines.

Examining MHAs themselves, the current studies identified 6 core features for a transdiagnostic therapy-accompanying app: mood checks, template library, reminders, goals and tasks, emergency kit, and questionnaire manager. Considering patients’ perspectives from previous research, they also reported similar core features for MHAs [10,47,62,63]. Psychotherapists highlighted accessibility, design, and customizability as important app characteristics. Consistent with previous research, accessibility remains a key characteristic of MHAs for both patients and psychotherapists [45,63,64]. Psychotherapists in the current studies highlighted the interrelatability of the app Steps. They found it helpful to have complete access to adolescents’ entries, as well as the opportunity to provide additional content. Moreover, accessibility for adolescents was also mentioned. As Steps was available only for Android-based smartphones, the operating system was frequently cited as a key exclusion factor. Across all 3 studies, the impact of app design on both adolescents’ and psychotherapists’ engagement was particularly emphasized. MHAs need to be designed to be motivating and incentivizing to ensure long-term use for therapy support [62]. Another core app characteristic mentioned in every study was the customizability of MHAs. For example, psychotherapists in study 3 highlighted that digitizing their therapy materials was an important factor in their use of Steps. They appreciated the ability to continue working with their own materials rather than being restricted to content provided by the app. Additionally, a high degree of customizability allows psychotherapists to use an app independently of their specific psychotherapeutic approaches. Most available MHAs are based on cognitive behavioral theory [18], which creates higher barriers to adoption for psychotherapists who align with other therapeutic approaches [25,55,61,65].

Limited experience with MHAs is not only a result of psychotherapists’ behavior but also a significant barrier to their integration into daily routines. Previous research suggests that limited experience with e-mental health is often accompanied by a lack of competency and only moderate knowledge of the advantages and possibilities of MHAs in psychotherapy. Consequently, limited experience with MHAs can lead to increased aversion toward their use [9,24,25,38,39,45,46]. In line with this, the majority of psychotherapists in studies 2 and 3 reported that they had never used or recommended MHAs before. Moreover, the stated requirements for change—*becoming aware of benefits* and *ease of use*—were reported multiple times in every study as key factors for MHA use. Consistent with the LAMH model of Feijt et al [42], aspects of ease of use were the most frequently mentioned in the current studies. All participants emphasized that MHAs need to be easy to understand and intuitive to use. Psychotherapists specifically highlighted the need for an app that is both easy to understand and simple to use, as daily routines leave little capacity for intensive familiarization with new methods such as MHAs. In Germany, the use of MHAs for psychotherapeutic treatment is rarely reimbursed by health insurance companies [66]. That may also contribute to the low willingness to engage with MHAs. Other important influencing factors were related to perceived benefits, such as “improvement of treatment quality” as a driver and “being unconvinced of benefits” as a barrier. In accordance with previous research, psychotherapists need to be convinced of the advantages of MHA use for both patients and their own workflow [8,24,39,45,47]. Psychotherapists reported being more likely to adopt MHAs if the app facilitated their working processes, particularly the organizational tasks of psychotherapy. Additionally, fitting the app to patients’ needs and maintaining face-to-face contact were also identified as general characteristics in the current studies. From the psychotherapists’ perspective, the usefulness of an app depends on the clients’ needs and the phase of psychotherapy. Particularly during the initial stages, when establishing a therapeutic relationship, psychotherapists would not introduce a therapy-accompanying app. They believe that building a strong relationship first is essential for the successful implementation of MHAs. This aligns with previous studies indicating that psychotherapeutic support is a crucial factor in patients’ engagement with apps [8].

Beyond various personal factors influencing psychotherapists’ use of MHAs, the current studies also highlighted organizational factors. One significant and common barrier was technological issues. Consistent with previous research, adequate technical infrastructure—such as a stable internet connection and proper devices—is essential for the adoption of MHAs [9,25,46]. In studies 2 and 3, fewer than half of the psychotherapists reported using a smartphone or tablet at work. Limited access to technical equipment may contribute to the perceived difficulty of using MHAs. Furthermore, in line with previous research, psychotherapists expressed concerns about data security [24,39,44,45]. Their concerns extended beyond data protection within the apps to include transparent clinical standards and guiding principles as essential requirements for MHA use in psychotherapy [24,25,39,45,48]. Although apps are increasingly being implemented in health care and supported by policy makers [66], many psychotherapists still do not feel sufficiently confident in using MHAs [9,44,62]. Additionally, in our study, psychotherapists reported uncertainties regarding legal provisions and responsibilities, such as those related to an optional communication feature or the provision of digitized diagnostic questionnaires. Additionally, the need for external triggers was mentioned as a requirement. In line with previous research, participating psychotherapists suggested features such as a reminder function in the therapist version of MHAs to enhance engagement [24,25]. Furthermore, the perceived commitment of the entire organization and management was highlighted as an important motivational factor for successfully and sustainably integrating MHAs into daily routines.

### Practical Implications and Future Research

The study results provide insights into which app features and characteristics are essential for psychotherapists in different psychotherapeutic contexts. Future app development should build on these findings and ensure that essential app features and characteristics are incorporated into MHAs. Designing MHAs based on psychotherapists’ needs will facilitate their integration into daily routines. App developers must strike a balance between maximum customizability and ease of use. For example, MHAs could offer a wide selection of templates and therapy materials through a library feature, along with the option to digitize psychotherapists’ own materials to expand the existing library. However, future research and app development should not focus solely on psychotherapists’ perspectives and needs but should also consider the perspectives and needs of adolescents. Moreover, the integration of MHAs into daily routines is influenced by various drivers and barriers. The most significant barriers to integrating MHAs were technological issues and conditions in daily practice, such as a lack of time and resources. The most important driver was the perceived improvement in treatment quality. To successfully integrate MHAs, implementation strategies need to be adjusted. The LAMH model of Feijt et al [42] provides a useful framework for understanding common drivers, barriers, general characteristics, and requirements for change, which were supported by our study results. These identified drivers and barriers must be directly addressed in implementation strategies. Psychotherapists who have not previously worked with MHAs require close support to implement app-based services into their daily treatment routines. Additionally, organizational changes are necessary to successfully integrate MHAs into the health care system. Psychotherapists need sufficient time and resources to familiarize themselves with MHAs and learn how to incorporate them into their practice. Moreover, adequate technical equipment and stable internet connections are essential for the effective integration of MHAs.

### Strengths and Limitations

The current studies have several strengths and limitations. One of the most significant strengths is the exploration of psychotherapists’ perspectives while they actively tested a transdiagnostic, therapy-accompanying app in their regular treatments. This approach allowed psychotherapists to go beyond theoretical considerations of potential drivers, barriers, or necessary app features. Instead, they gained firsthand experience with MHAs, identifying which features they found helpful and which were lacking in their work. Furthermore, to the best of our knowledge, this is the first study exploring the use of MHAs in the inpatient treatment of adolescents. However, the present studies also have some limitations. The small sample sizes in all 3 studies suggest that the app Steps was not well implemented in practice, limiting the generalizability of the findings to other settings. Additionally, the small sample sizes and the recruitment method pose a risk of selection bias. For instance, psychotherapists with a greater interest in technological developments may have been more likely to participate in the studies.

### Conclusions

In sum, psychotherapists in this study were generally open to therapy-accompanying MHAs. However, both study participation and app usage were low. Psychotherapists identified various drivers and barriers influencing their use of MHAs, highlighting that adoption is shaped not only by personal factors but also by organizational challenges. To enable psychotherapists to integrate MHAs into their practice, changes in current working conditions are necessary. For example, they require adequate technical equipment and sufficient time to familiarize themselves with new therapy methods such as MHAs. Successfully integrating MHAs into daily psychotherapeutic routines is a challenge for the entire health care system, not just for individual psychotherapists.

## Appendix

Multimedia Appendix 1 List of surveys presented separately for each study.

Multimedia Appendix 2 The final set of categories for the deductive qualitative content analysis.

Multimedia Appendix 3 A flowchart of study participation is presented for each study, along with the frequencies of self-created questions regarding the technical equipment available at work, the use of media and smartphone apps, and the integration of media in psychotherapies for studies 2 and 3.

## Abbreviations

ATI: Affinity for Technology Interaction
COREQ: Consolidated Criteria for Reporting Qualitative Research
LAMH: Levels of Adoption of e-Mental Health
MTPS: Psychotherapists’ Attitudes Toward Using Modern Technologies in Psychotherapy and Counselling Scale
TA-EG: Affinity for Technology
uMARS: user version of the Mobile Application Rating Scale
UTAUT: Unified Theory of Acceptance and Usability of Technology
WHO: World Health Organization

## Data Availability

The data sets generated or analyzed during this study are available from the corresponding author upon reasonable request.
