# Supplementary material for: The Use of Mobile Apps in Adolescent Psychotherapy: Assessment of Psychotherapists’ Perspectives
Source: JMIR Form Res. 2025 Apr 8;9:e65788. doi: 10.2196/65788 (PMC12015344; doi:10.2196/65788)
Supplement: Multimedia Appendix 1 [file formative_v9i1e65788_app1.pdf]

Multimedia Appendix 1

List of surveys presented separately for each study

1.1 Study one: Inpatient treatment of adolescents 2
1.1.1 Psychotherapists – Baseline survey 2
1.1.2 Psychotherapists – Post-survey 6
1.1.3 Other employees – survey 9
1.2 Study two: Psychiatric outpatient clinics 15
1.2.1 Psychotherapists – Baseline survey 15
1.2.2 Psychotherapists – Post-survey 19
1.3 Study three: Psychotherapy in private practices 26
1.3.1 Psychotherapists – Baseline survey 26
1.3.2 Psychotherapists – Post-survey 32

The original surveys were in German. For this publication, the surveys were translated in English.

1.1 Study one: Inpatient treatment of adolescents

1.1.1 Psychotherapists – Baseline survey

Soziodemografie / Sociodemographics

Zunächst ein paar Fragen zu Ihrer Person. / First of all, a few questions about yourself.

Wie alt bist du? ___________ Jahre

How old are you? ___________ Years

Welche Berufsqualifikation hast du? / What professional qualification do you have?

- ☐ Kinder- und Jugendpsychotherapeut:in in Ausbildung / Child and adolescent psychotherapist in training
- ☐ Kinder- und Jugendpsychotherapeut:in / Child and adolescent psychotherapist:in
- ☐ Assistenzarzt:Assistenzärztin / Assistant doctor
- ☐ Arzt:Ärztin / Doctor

Wie viel Berufserfahrung hast du bereits? ___________ Jahre ___________ Monate

How much work experience do you already have? ___________ years ___________ months

TA-EG (Karrer et al., 2009)

Als nächstes möchten wir deine persönliche Meinung und Erfahrungen zu verschiedenen Aspekten elektronischer Geräte und deinem Umgang mit diesen erfassen. Unter dem Begriff „elektronische Geräte“ verstehen wir Geräte, wie Computer, Handys, Digitalkameras, DVD- oder Mp3-Spieler, Geld- oder Ticketautomaten oder neue Systeme im Auto (z.B. Navigationssysteme). Nicht gemeint sind Werkzeuge, Haushaltsgeräte, Fahrzeuge oder Fahrzeugmotoren. Bitte gib bei jeder der folgenden Aussagen an, wie gut sie auf dich persönlich zutrifft. Kreuze dazu auf der rechten Seite das Feld an, das deiner Meinung am besten entspricht.

Next, we would like to gather your personal opinions and experiences on various aspects of electronic devices and how you use them. By the term "electronic devices" we mean devices such as computers, cell phones, digital cameras, DVD or MP3 players, cash or ticket machines or new systems in cars (e.g. navigation systems). This does not include tools, household appliances, vehicles or vehicle engines. For each of the following statements, please indicate how well it applies to you personally. To do this, tick the box on the right-hand side that best corresponds to your opinion.

		Trifft gar nicht zu<br>Does not apply at all	trifft eher nicht zu<br>Rather not true	Trifft weder noch<br>Neither applies nor applies	trifft eher zu<br>Rather true	trifft voll zu<br>Fully applies
1.	Ich liebe es, neue elektronische Geräte zu besitzen.<br>I love owning new electronic devices.	☐	☐	☐	☐	☐
2.	Elektronische Geräte machen krank.<br>Electronic devices make you ill.	☐	☐	☐	☐	☐
3.	Ich gehe gern in den Fachhandel für elektronische Geräte.<br>I like going to specialist shops for electronic devices.	☐	☐	☐	☐	☐
4.	Ich habe bzw. hätte Verständnisprobleme beim Lesen von Elektronik- und Computerzeitschriften.<br>I have or would have problems understanding electronic and computer magazines.	☐	☐	☐	☐	☐
5.	Elektronische ermöglichen einen hohen Lebensstandard.<br>Electronic devices enable a high standard of living.	☐	☐	☐	☐	☐
6.	Elektronische Geräte führen zu geistiger Verarmung.<br>Electronic devices lead to mental impoverishment.	☐	☐	☐	☐	☐
7.	Elektronische Geräte machen vieles umständlicher.<br>Electronic devices make many things more complicated.	☐	☐	☐	☐	☐
8.	Ich informiere mich über elektronische Geräte, auch wenn ich keine Kaufabsicht habe.<br>I inform myself about electronic devices, even if I have no intention of buying them.	☐	☐	☐	☐	☐
9.	Elektronische Geräte machen unabhängig.<br>Electronic devices make me independent	☐	☐	☐	☐	☐
10.	Es macht mir Spaß, ein elektronisches Gerät auszuprobieren.<br>I enjoy trying out an electronic device.	☐	☐	☐	☐	☐
11.	Elektronische Geräte erleichtern mir den Alltag.<br>Electronic devices make my everyday life easier.	☐	☐	☐	☐	☐
12.	Elektronische Geräte erhöhen die Sicherheit.<br>Electronic devices increase safety.	☐	☐	☐	☐	☐
13.	Elektronische Geräte verringern den persönlichen Kontakt zwischen den Menschen.<br>Electronic devices reduce personal contact between people.	☐	☐	☐	☐	☐
14.	Ich kenne die meisten Funktionen der elektronischen Geräte, die ich besitze.<br>I know most of the functions of the electronic devices I own.	☐	☐	☐	☐	☐
15.	Ich bin begeistert, wenn ein neues elektronisches Gerät auf den Markt kommt.<br>I am excited when a new electronic device comes onto the market.	☐	☐	☐	☐	☐
16.	Elektronische Geräte verursachen Stress.<br>Electronic devices cause stress.	☐	☐	☐	☐	☐
17.	Ich kenne mich im Bereich elektronischer Geräte aus.<br>I know my way around electronic devices.	☐	☐	☐	☐	☐

18.	Es fällt mir leicht, die Bedienung eines elektronischen Geräts zu lernen.<br>It is easy for me to learn how to operate an electronic device.	☐	☐	☐	☐	☐
19.	Elektronische Geräte helfen, an Informationen zu gelangen.<br>Electronic devices help me to access information.	☐	☐	☐	☐	☐

Akzeptanz der App *Steps* / Acceptance of the app *Steps*

UTAUT (angelehnt an die Theorie von Venkatesh et al.; Die Items sind selbst erstellt und auf den stationären Kontext angepasst.)

UTAUT (based on the theory of Venkatesh et al.; The items are self-created and adapted to the inpatient context.)

Im nächsten Schritt geht es um Ihre Erwartungen zur Nutzung von *Steps*. Geben sie bitte an, wie sehr Sie den folgenden Aussagen zustimmen.

The next step is about your expectations regarding the use of *Steps*. Please indicate how much you agree with the following statements.

		Stimme gar nicht zu<br>Totally disagree	Stimme eher nicht zu<br>Rather disagree	Weder noch<br>Neither	Stimme eher zu<br>Agree somewhat	Stimme voll zu<br>Totally agree
1.	*Steps* wird die Qualität der stationären Therapie unserer Patient:innen verbessern.<br>*Steps* will improve the quality of inpatient therapy for our patients.	☐	☐	☐	☐	☐
2.	Durch den Einsatz von *Steps* wird unsere Arbeit erleichtert.<br>The use of *Steps* will make our work easier.	☐	☐	☐	☐	☐
3.	*Steps* wird sich gut in unseren Stationsalltag integrieren lassen.<br>"Steps will be easy to integrate into our daily work routine.	☐	☐	☐	☐	☐
4.	Ich glaube, dass *Steps* eine gute Entwicklung für unsere Station ist.<br>I believe that *Steps* is a good development for our ward.	☐	☐	☐	☐	☐
5.	*Steps* wird hilfreich für unsere Patient:innen sein.<br>*Steps* will be helpful for our patients.	☐	☐	☐	☐	☐

6.	Ich werde es einfach finden „Stpes“ zu verstehen.<br><br>I will find it easy to understand *Steps*.	☐	☐	☐	☐	☐
7.	Es wird mir leichtfallen, Patient:innen die App zu erklären.<br><br>It will be easy for me to explain the app to patients.	☐	☐	☐	☐	☐
8.	*Steps* wird unseren Stationsalltag verkomplizieren.<br><br>*Steps* will complicate our daily ward routine.	☐	☐	☐	☐	☐
9.	Ich fühle mich gut vorbereitet auf die Einführung von *Steps*.<br><br>I feel well prepared for the introduction of *Steps*.	☐	☐	☐	☐	☐
10.	Ich habe ausreichendes Material erhalten, um mich auf die Einführung von *Steps* vorzubereiten.<br><br>I have received sufficient material to prepare me for the introduction of *Steps*.	☐	☐	☐	☐	☐
11.	*Steps* kann ich mit meinen Smartphone- und Computer-Kenntnissen nutzen.<br><br>I can use *Steps* with my smartphone and computer skills.	☐	☐	☐	☐	☐
12.	Ich freue mich auf die Einführung von *Steps*.<br><br>I am looking forward to the introduction of *Steps*.	☐	☐	☐	☐	☐

Worauf freust du dich bei *Steps* am meisten? / What are you most looking forward to with *Steps*?

____

Was sind Befürchtungen, die du bezüglich *Steps* hast? / What fears do you have about *Steps*?

____

Was ist für dich wahrscheinlich die größte Herausforderung bei der Nutzung von *Steps*? /
What is probably the biggest challenge for you when using *Steps*?

____

Möchtest du uns noch etwas Anderes mitteilen? / Is there anything else you would like to tell us?

____

1.1.2 Psychotherapists – Post-survey

Akzeptanz der App *Steps* / Acceptance of the app *Steps*

UTAUT (angelehnt an die Theorie von Venkatesh et al.)

UTAUT (based on the theory of Venkatesh et al.)

Zunächst möchten wir von dir erfahren, wie du die Arbeit mit *Steps* wahrgenommen hast. Kreuze bitte an, wie sehr die folgenden Aussagen auf dich zutreffen.

First of all, we would like to find out how you perceived working with *Steps*. Please mark how much the following statements apply to you.

		Stimme gar nicht zu<br>Totally disagree	Stimme eher nicht zu<br>Rather disagree	Weder noch<br>Neither	Stimme eher zu<br>Agree somewhat	Stimme voll zu<br>Totally agree
1.	Es macht Spaß *Steps* zu nutzen.<br>It is fun to use *Steps*.	☐	☐	☐	☐	☐
2.	*Steps* erleichtert meine therapeutische Arbeit.<br>*Steps* makes my therapeutic work easier.	☐	☐	☐	☐	☐
3.	Ich finde *Steps* für meine therapeutische Arbeit nützlich.<br>I find *Steps* useful for my therapeutic work.	☐	☐	☐	☐	☐
4.	Den Umgang mit *Steps* zu lernen, war einfach für mich.<br>It was easy for me to learn how to use *Steps*.	☐	☐	☐	☐	☐
5.	Ich brauche mehr Zeit für meine Arbeit, wenn ich *Steps* nutze.<br>I need more time for my work when I use *Steps*.	☐	☐	☐	☐	☐
6.	Ich habe das technische Wissen, um *Steps* zu nutzen.<br>I have the technical knowledge to use *Steps*.	☐	☐	☐	☐	☐
7.	Es fällt mir leicht, *Steps* für meine therapeutische Arbeit zu nutzen.<br>It is easy for me to use *Steps* for my therapeutic work.	☐	☐	☐	☐	☐

8.	**Ich glaube, dass *Steps* gut zu meiner Art zu arbeiten passt.**<br><br>**I believe that *Steps* fits well with my way of working.**	☐	☐	☐	☐	☐
9.	*Steps* steigert die Qualität meiner Arbeit.<br><br>*Steps* increases the quality of my work.	☐	☐	☐	☐	☐
10.	Ich habe die benötigten Ressourcen, um *Steps* gut nutzen zu können.<br><br>I have the resources I need to make good use of *Steps*.	☐	☐	☐	☐	☐

Zufriedenheit von *Steps* / Satisfaction of *Steps*

(selbst erstellte Items) / (self-created items)

Nun gib bitte an, wie zufrieden du mit der App bist. Kreuze an, wie sehr die folgenden Aussagen auf dich zutreffen.

Now please indicate how satisfied you are with the app. Mark how much the following statements apply to you.

		Trifft gar nicht zu<br><br>Does not apply at all	Trifft kaum zu<br><br>Hardly true	Trifft eher zu<br><br>Rather true	Trifft voll und ganz zu<br><br>Completely true
1.	*Steps* hat eine hohe Qualität.<br>*Steps* has a high quality.	☐	☐	☐	☐
2.	*Steps* entspricht meinen Bedürfnissen.<br>*Steps* meets my needs.	☐	☐	☐	☐
3.	Ich würde einem*einer Kolleg*in *Steps* weiterempfehlen.<br>I would recommend *Steps* to a colleague.	☐	☐	☐	☐
4.	Im Großen und Ganzen bin ich mit *Steps* zufrieden.<br>Overall, I am satisfied with *Steps*.	☐	☐	☐	☐
5.	Die Therapeut*innen-Ansicht ist einfach zu verstehen.<br>The therapist view is easy to understand.	☐	☐	☐	☐
6.	Das Design der App gefällt mir gut.<br>I like the design of the app.	☐	☐	☐	☐
7.	Die grafische Aufmachung der Therapeut*innen-Ansicht ist gut gelungen.<br>The graphic design of the therapist view is well done.	☐	☐	☐	☐
8.	Die Nutzung von *Steps* passt gut in den Stationsalltag.<br>The use of *Steps* fits well into everyday life on the ward.	☐	☐	☐	☐

Welche Funktionen der App haben dir in deiner therapeutischen Arbeit nicht geholfen? / Which functions of the app have not helped you in your therapeutic work?

___

Welche Funktionen haben dir bei deiner Arbeit gefehlt? / What functions did you miss in your work?

___

Was gefällt dir an *Steps* besonders gut? / What do you particularly like about *Steps*?

___

Wo siehst du bei "Steps" Verbesserungspotential? / Where do you see potential for improvement in *Steps*?

___

Möchtest du uns noch etwas Anderes mitteilen? / Is there anything else you would like to tell us?

___

Würdest du anderen Therapeut*innen *Steps* weiterempfehlen? / Would you recommend *Steps* to other therapists?

☐ ja / yes ☐ nein / no

Wenn man *Steps* als Therapie-Tool kaufen könnte, wie viel würdest du dafür bezahlen? /

If you could buy *Steps* as a therapy tool, how much would you pay for it?

_____________ Euro / Euro

1.1.3 Other employees – survey

Soziodemografie / Sociodemographics

Zunächst haben wir ein paar Fragen zu deiner Person:

First, we have a few questions about you:

1. Wie alt bist du? ____________ Jahre
   How old are you? ____________ years

2. Welchem Geschlecht fühlst du dich zugehörig?
   - ☐ weiblich
   - ☐ männlich
   - ☐ divers

   Which gender do you feel you belong to?
   - ☐ female
   - ☐ male
   - ☐ other

3. Zu welcher Berufsgruppe gehörst du? / Which professional group do you belong to?
   - ☐ Auszubildende:r: / Apprentice ______________________________
   - ☐ Heilerziehungspfleger:in / Curative educator
   - ☐ Erzieher:in / Educator
   - ☐ Krankenpfleger:in / Nurse
   - ☐ Fachtherapeut:in / Specialist therapist
   - ☐ Anderes: / Other: ____

4. Wie viel Berufserfahrung hast du bereits? ____________ Jahre ____________ Monate
   How much work experience do you already have? ____________ years ____________ months

5. Bist du Primary Nurse? Are you a primary nurse?
   - ☐ ja / yes
   - ☐ nein / no

TA-EG (Karrer et al., 2009)

Als nächstes möchten wir deine persönliche Meinung und Erfahrungen zu verschiedenen Aspekten elektronischer Geräte und deinem Umgang mit diesen erfassen. Unter dem Begriff „elektronische Geräte“ verstehen wir Geräte, wie Computer, Handys, Digitalkameras, DVD- oder Mp3-Spieler, Geld- oder Ticketautomaten oder neue Systeme im Auto (z.B. Navigationssysteme). Nicht gemeint sind Werkzeuge, Haushaltsgeräte, Fahrzeuge oder Fahrzeugmotoren. Bitte gib bei jeder der folgenden Aussagen an, wie gut sie auf dich persönlich zutrifft. Kreuze dazu auf der rechten Seite das Feld an, das deiner Meinung am besten entspricht.

Next, we would like to gather your personal opinions and experiences on various aspects of electronic devices and how you use them. By the term "electronic devices" we mean devices such as computers, cell phones, digital cameras, DVD or MP3 players, cash or ticket machines or new systems in cars (e.g. navigation systems). Tools, household appliances, vehicles or vehicle engines are not meant. For each of the following statements, please indicate how well it applies to you personally. To do this, tick the box on the right-hand side that best corresponds to your opinion.

		Trifft gar nicht zu<br>Does not apply at all	trifft eher nicht zu<br>Rather not true	Trifft weder noch<br>Neither applies nor applies	trifft eher zu<br>Rather true	trifft voll zu<br>Fully applies
1.	Ich liebe es, neue elektronische Geräte zu besitzen.<br>I love owning new electronic devices.	☐	☐	☐	☐	☐
2.	Elektronische Geräte machen krank.<br>Electronic devices make you ill.	☐	☐	☐	☐	☐
3.	Ich gehe gern in den Fachhandel für elektronische Geräte.<br>I like going to specialist shops for electronic devices.	☐	☐	☐	☐	☐
4.	Ich habe bzw. hätte Verständnisprobleme beim Lesen von Elektronik- und Computerzeitschriften.<br>I have or would have problems understanding electronic and computer magazines.	☐	☐	☐	☐	☐
5.	Elektronische ermöglichen einen hohen Lebensstandard.<br>Electronic devices enable a high standard of living.	☐	☐	☐	☐	☐
6.	Elektronische Geräte führen zu geistiger Verarmung.<br>Electronic devices lead to mental impoverishment.	☐	☐	☐	☐	☐
7.	Elektronische Geräte machen vieles umständlicher.<br>Electronic devices make many things more complicated.	☐	☐	☐	☐	☐
8.	Ich informiere mich über elektronische Geräte, auch wenn ich keine Kaufabsicht habe.<br>I inform myself about electronic devices, even if I have no intention of buying them.	☐	☐	☐	☐	☐
9.	Elektronische Geräte machen unabhängig.<br>Electronic devices make me independent	☐	☐	☐	☐	☐
10.	Es macht mir Spaß, ein elektronisches Gerät auszuprobieren.<br>I enjoy trying out an electronic device.	☐	☐	☐	☐	☐
11.	Elektronische Geräte erleichtern mir den Alltag.<br>Electronic devices make my everyday life easier.	☐	☐	☐	☐	☐

12.	Elektronische Geräte erhöhen die Sicherheit.<br>Electronic devices increase safety.	☐	☐	☐	☐	☐
13.	Elektronische Geräte verringern den persönlichen Kontakt zwischen den Menschen.<br>Electronic devices reduce personal contact between people.	☐	☐	☐	☐	☐
14.	Ich kenne die meisten Funktionen der elektronischen Geräte, die ich besitze.<br>I know most of the functions of the electronic devices I own.	☐	☐	☐	☐	☐
15.	Ich bin begeistert, wenn ein neues elektronisches Gerät auf den Markt kommt.<br>I am excited when a new electronic device comes onto the market.	☐	☐	☐	☐	☐
16.	Elektronische Geräte verursachen Stress.<br>Electronic devices cause stress.	☐	☐	☐	☐	☐
17.	Ich kenne mich im Bereich elektronischer Geräte aus.<br>I know my way around electronic devices.	☐	☐	☐	☐	☐
18.	Es fällt mir leicht, die Bedienung eines elektronischen Geräts zu lernen.<br>It is easy for me to learn how to operate an electronic device.	☐	☐	☐	☐	☐
19.	Elektronische Geräte helfen, an Informationen zu gelangen.<br>Electronic devices help me to access information.	☐	☐	☐	☐	☐

Akzeptanz der App *Steps* / Acceptance of the app *Steps*

UTAUT (based on the theory of Venkatesh et al.)

Nun möchten wir von dir wissen, wie du die App *Steps* wahrgenommen hast.

Now we would like to know how you perceived the *Steps* app.

		Stimme gar nicht zu<br>Totally disagree	Stimme eher nicht zu<br>Rather disagree	Weder noch<br>Neither	Stimme eher zu<br>Agree somewhat	Stimme voll zu<br>Totally agree
1.	*Steps* wird die Qualität der stationären Therapie unserer Patient:innen verbessern.<br>*Steps* improves the quality of inpatient therapy for our patients.	☐	☐	☐	☐	☐
2.	Durch den Einsatz von *Steps* wird unsere Arbeit erleichtert.<br>The use of *Steps* will make our work easier.	☐	☐	☐	☐	☐

3.	*Steps* wird sich gut in unseren Stationsalltag integrieren lassen.<br><br>*Steps* integrates well into our daily ward routine.	☐	☐	☐	☐	☐
4.	Ich glaube, dass *Steps* eine gute Entwicklung für unsere Station ist.<br><br>I believe that *Steps* is a good development for our ward.	☐	☐	☐	☐	☐
5.	*Steps* wird hilfreich für unsere Patient:innen sein.<br><br>*Steps* is helpful for our patients	☐	☐	☐	☐	☐
6.	Ich werde es einfach finden „Stpes“ zu verstehen.<br><br>I find it easy to understand *Steps*.	☐	☐	☐	☐	☐
7.	Es wird mir leichtfallen, Patient:innen die App zu erklären.<br><br>It's easy for me to explain the app to patients.	☐	☐	☐	☐	☐
8.	*Steps* wird unseren Stationsalltag verkomplizieren.<br><br>*Steps* complicates our daily ward routine.	☐	☐	☐	☐	☐
9.	Ich fühle mich gut vorbereitet auf die Einführung von *Steps*.<br><br>I feel well informed about *Steps*.	☐	☐	☐	☐	☐
10.	Ich habe ausreichendes Material erhalten, um mich auf die Einführung von *Steps* vorzubereiten.<br><br>I have received sufficient material to prepare me for the introduction of *Steps*.	☐	☐	☐	☐	☐
11.	*Steps* kann ich mit meinen Smartphone- und Computer-Kenntnissen nutzen.<br><br>I can use *Steps* with my smartphone and computer skills.	☐	☐	☐	☐	☐
12.	Ich freue mich auf die Einführung von *Steps*.<br><br>I enjoy working with *Steps*.	☐	☐	☐	☐	☐

Veränderungen durch *Steps* / Changes through *Steps*

Als nächstes möchten wir von dir noch etwas mehr darüber erfahren, welche Veränderung-gen du durch die Einführung von *Steps* und den Stations-Smartphones wahrgenommen hast.

Next, we would like to find out a little more about the changes you have noticed as a result of the introduction of *Steps* and the Stations smartphones.

Hat sich an eurer Beziehungsarbeit zu den Patient:innen durch die Einführung der Stations-Smartphones etwas geändert? / Has the introduction of clinic-owned smartphones changed anything in your relationship work with patients?

☐ ja / yes ☐ nein / no

Wenn ja: Was hat sich geändert? / If so, what has changed?

____

Hat sich der Stationsalltag durch *Steps* oder die Stationssmartphones geändert? / Has everyday life in the station changed as a result of *Steps* or the station smartphones?

☐ ja / yes ☐ nein / no

Wenn ja: Was hat sich geändert? / If so, what has changed?

____

Hat sich eure Arbeitsweise durch die Stationssmartphones oder *Steps* geändert? / Has the station smartphones or *Steps* changed the way you work?

☐ ja / yes ☐ nein / no

Wenn ja: Was hat sich geändert? / If so, what has changed?

____

Offenes Feedback / Open feedback

Was gefällt dir an *Steps* besonders gut? / What do you particularly like about *Steps*?

____

Was gefällt dir an *Steps* nicht? / What don't you like about *Steps*?

____

Wo siehst du bei “Steps” Verbesserungspotential? / Where do you see potential for improvement in *Steps*?

Wie würdest du dir in Zukunft den Einsatz von *Steps* auf eurer Station wünschen? / How would you like to see *Steps* used on your ward in the future?

Möchtest du uns noch etwas Anderes mitteilen? / Is there anything else you would like to tell us?

1.2 Study two: Psychiatric outpatient clinics

1.2.1 Psychotherapists – Baseline survey

Soziodemografie / Sociodemographics

Zunächst ein paar Fragen zu Ihrer Person. / First of all, a few questions about yourself.

Standort: / Location:

- ☐ Bielefeld / Bielefeld
- ☐ Mannheim / Mannheim
- ☐ Frankfurt / Frankfurt
- ☐ Köln / Cologne

Alter: ___________ Jahre

Age: ___________ years

Berufserfahrung: ___________ Jahre ___________ Monate

Professional experience: ___________ years ___________ months

Berufsqualifikation

Professional qualification

- ☐ (Kinder- und Jugend-) Psychotherapeut:in in Ausbildung
  (Child and adolescent) psychotherapist in training
- ☐ (Kinder- und Jugend-) Psychotherapeut:in
  (Child and adolescent) psychotherapist
- ☐ Assistenzärzt:in
  Assistant doctor
- ☐ Fachärzt:in für (Kinder- und Jugend-)psychiatrie und Psychotherapie
  Specialist in (child and adolescent) psychiatry and psychotherapy
- ☐ Anderes: / Other _______

Medienaffinität: Besitz und Nutzung digitaler Medien

Media affinity: Ownership and use of digital media

Als nächstes möchten wir von Ihnen erfahren, ob und wie Sie digitale Medien beruflich nutzen.

Next, we would like to find out whether and how you use digital media at work.

Besitzen Sie ... ein Smartphone? ☐ Ja ☐ Nein

Do you own ... a smartphone? ☐ Yes ☐ No

Besitzen Sie ... einen Computer? ☐ Ja ☐ Nein

Do you own ... a computer? ☐ Yes ☐ No

Besitzen Sie ... einen Laptop? ☐ Ja ☐ Nein

Do you own ... a laptop? ☐ Yes ☐ No

Besitzen Sie ... ein Tablet? ☐ Ja ☐ Nein

Do you own ... a tablet? ☐ Yes ☐ No

In Ihrem Alltag: Wie viel Zeit am Tag nutzen Sie durchschnittlich Ihr Smartphone oder Computer/Laptop/Tablet?

☐ 0-2 Stunden
☐ 2-4 Stunden
☐ 4-6 Stunden
☐ 6-8 Stunden
☐ mehr als 8 Stunden

In your everyday life: How much time a day do you use your smartphone or computer/laptop/tablet on average?

☐ 0-2 hours
☐ 2-4 hours
☐ 4-6 hours
☐ 6-8 hours
☐ more than 8 hours

Nutzen Sie digitale Medien während Ihrer Sitzungen? / Do you use digital media during the therapy sessions?

☐ Ja / Yes ☐ Nein / No

Wenn ja: Welche digitalen Medien nutzen Sie? / If yes: Which digital media do you use?

____

Nutzen Sie Apps für Ihre Behandlung? / Do you use apps for your treatment?

☐ Ja / Yes ☐ Nein / No

Wenn ja: Welche Apps nutzen Sie? / If yes: Which apps do you use?

Wenn ja: Haben Sie schon einmal eine DiGA (Digitale Gesundheitsanwendung) verschrieben? / If yes: Have you ever prescribed a DiGA (digital health application)?

☐ Ja / Yes ☐ Nein / No

Erfassen Sie während der Probatorik Angaben zur Mediennutzung von Kindern und Jugendlichen? / Do you collect data on the media use of children and adolescents during the diagnostics?

☐ nie / never
☐ manchmal / sometimes
☐ häufig / often
☐ immer / always

Voreinstellungen zur Nutzung einer psychotherapeutischen App

UTAUT (angelehnt an die Theorie von Venkatesh et al.)

Preferences for the use of a psychotherapeutic app

UTAUT (based on the theory of Venkatesh et al.)

Im nächsten Schritt geht es um Ihre Erwartungen zur Nutzung von *Steps*. Geben sie bitte an, wie sehr Sie den folgenden Aussagen zustimmen.

The next step is about your expectations regarding the use of *Steps*. Please indicate how much you agree with the following statements.

		Stimme gar nicht zu Totally disagree	Stimme eher nicht zu Rather disagree	Weder noch Neither	Stimme eher zu Tend to agree	Stimme voll zu Fully agree
1	Ich werde Spaß haben *Steps* zu nutzen. I will have fun using *Steps*.	☐	☐	☐	☐	☐

2	*Steps* wird meine therapeutische Arbeit erleichtern.<br>*Steps* will make my therapeutic work easier.	☐	☐	☐	☐	☐
3	Ich werde *Steps* für meine therapeutische Arbeit nützlich finden.<br>I will find *Steps* useful for my therapeutic work.	☐	☐	☐	☐	☐
4	Den Umgang mit *Steps* zu lernen, wird einfach für mich.<br>Learning how to use *Steps* will be easy for me.	☐	☐	☐	☐	☐
5	Ich werde mehr Zeit für meine Arbeit brauchen, wenn ich *Steps* nutze.<br>I will need more time for my work if I use *Steps*.	☐	☐	☐	☐	☐
6	Ich habe das technische Wissen, um *Steps* nutzen zu können.<br>I have the technical knowledge to be able to use *Steps*.	☐	☐	☐	☐	☐
7	Es wird mir leichtfallen, *Steps* für meine therapeutische Arbeit zu nutzen.<br>It will be easy for me to use *Steps* for my therapeutic work.	☐	☐	☐	☐	☐
8	Ich glaube, dass *Steps* gut zu meiner Art zu arbeiten passen wird.<br>I believe that *Steps* will fit in well with my way of working.	☐	☐	☐	☐	☐
9	*Steps* wird die Qualität meiner Arbeit steigern.<br>*Steps* will increase the quality of my work.	☐	☐	☐	☐	☐
10	Ich habe die benötigten Ressourcen, um *Steps* gut nutzen zu können.<br>I have the resources I need to make good use of *Steps*.	☐	☐	☐	☐	☐

1.2.2 Psychotherapists – Post-survey

Wahrgenommene App-Qualität / Perceived app quality

uMARS (Stoyanov et al. 2016)

In den letzten Monaten konnten Sie die therapieunterstützende, transdiagnostische App "Steps - Mein Weg zum Ziel" intensiv kennenlernen und nutzen. Wir möchten nun gerne Ihre Meinung zu dieser App hören. Während der Therapien hatten Sie die Möglichkeit sowohl mit der App-Version für Patient:innen zu arbeiten als auch mit der webbasierten Therapeut:innen-Version. Deshalb möchten wir von Ihnen gerne erfahren, wie Sie beide Anwendungsmöglichkeiten erlebt haben und wie Sie die Qualität beider Anwendungsversionen einschätzen. Überlegen Sie sich deshalb bitte für jede Aussage, welche Antwort auf die App-Version und welche Antwort auf die webbasierte Therapeut:innen-Version am besten passt. Wir sind an Ihrer ehrlichen Meinung interessiert. Es gibt kein richtig oder falsch.

Over the past few months, you have had the opportunity to familiarise yourself with and use the therapy-supporting, transdiagnostic app "Steps - My way to my goal". We would now like to hear your opinion on this app. During the therapy sessions, you had the opportunity to work with both the app version for patients and the web-based therapist version. We would therefore like to find out how you experienced both application options and how you rate the quality of both application versions. For each statement, please consider which answer best fits the app version and which answer best fits the web-based therapist version. We are interested in your honest opinion. There is no right or wrong.

1. **Es macht Spaß, die Anwendung zu benutzen. / it is fun to use the application.**

App-Version: / app version:
☐ Stimme gar nicht zu / totally disagree
☐ Stimme eher nicht zu / rather disagree
☐ weder noch / neither
☐ stimme eher zu / tend to agree
☐ stimme voll und ganz zu / fully agree

Web-Version: / web version:
☐ Stimme gar nicht zu / totally disagree
☐ Stimme eher nicht zu / rather disagree
☐ weder noch / neither
☐ stimme eher zu / tend to agree
☐ stimme voll und ganz zu / fully agree

Welche Funktionen oder Dinge der Anwendung sorgen dafür, dass es mehr Spaß macht die Anwendung zu verwenden? Zum Beispiel im Vergleich zu anderen ähnlichen Anwendungen? / What features or things about the application make it more fun to use? For example, compared to other similar applications?

App-Version: / app version:
____
____

Web-Version: / web version:
____
____

2. **Die Anwendung ist interessant zu benutzen. / It is an interesting application to use.**

App-Version: / app version:
□ Stimme gar nicht zu / totally disagree
□ Stimme eher nicht zu / rather disagree
□ weder noch / neither
□ stimme eher zu / tend to agree
□ stimme voll und ganz zu / fully agree

Web-Version: / web version:
□ Stimme gar nicht zu / totally disagree
□ Stimme eher nicht zu / rather disagree
□ weder noch / neither
□ stimme eher zu / tend to agree
□ stimme voll und ganz zu / fully agree

Welche Funktionen oder Dinge der Anwendung sorgen dafür, dass die Informationen in einer interessanten Weise präsentiert werden? Zum Beispiel im Vergleich zu anderen ähnlichen Anwendungen? / What features or things in the application ensure that the information is presented in an interesting way? For example, in comparison to other similar applications?

App-Version: / app version:
____
____

Web-Version: / web version:
____
____

3. Die Anwendung ist individuell anpassbar. Das bedeutet: Es ist möglich die Anwendung nach meinen eigenen Wünschen anzupassen (z.B. Töne, Benachrichtigungen oder Inhalte). Die Anpassungen werden gespeichert. Sie müssen nicht bei jeder Nutzung erneut eingestellt werden. / The application is customisable. This means: It is possible to customise the application according to my own wishes (e.g. sounds, notifications or content). The customisations are saved. They do not have to be reset each time they are used

App-Version: / app version:
□ Stimme gar nicht zu / totally disagree
□ Stimme eher nicht zu / rather disagree
□ weder noch / neither
□ stimme eher zu / tend to agree
□ stimme voll und ganz zu / fully agree

Web-Version: / web version:
□ Stimme gar nicht zu / totally disagree
□ Stimme eher nicht zu / rather disagree
□ weder noch / neither
□ stimme eher zu / tend to agree
□ stimme voll und ganz zu / fully agree

4. Die Anwendung ist interaktiv. Das bedeutet zum Beispiel: Ich kann eigene Inhalte eingeben, die Anwendung fordert mich zu Eingaben auf (z.B. durch Erinnerungen), ich erhalte Feedback durch die Anwendung oder ich habe die Möglichkeit mich über die Anwendung mit anderen auszutauschen. / The application is interactive. This means, for example: I can enter my own content, the application prompts me to make entries (e.g. through reminders), I receive feedback through the application or I have the opportunity to exchange information with others via the application.

App-Version: / app version:
☐ Stimme gar nicht zu / totally disagree
☐ Stimme eher nicht zu / rather disagree
☐ weder noch / neither
☐ stimme eher zu / tend to agree
☐ stimme voll und ganz zu / fully agree

Web-Version: / web version:
☐ Stimme gar nicht zu / totally disagree
☐ Stimme eher nicht zu / rather disagree
☐ weder noch / neither
☐ stimme eher zu / tend to agree
☐ stimme voll und ganz zu / fully agree

5.Die Anwendung ist zielgruppenspezifisch. Das bedeutet: Die App-Version bzw. die Web-Version ist, ihrer Zielgruppe entsprechend, ansprechend gestaltet. Die Anwendung hat eine die Zielgruppe ansprechende Optik und Sprache. Das Design ist für die Zielgruppe ansprechend. / The application is target group-specific. This means that the app version or the web version is designed to appeal to its target group. The application has a look and language that appeals to the target group. The design is appealing to the target group.

App-Version: / app version:
☐ Stimme gar nicht zu / totally disagree
☐ Stimme eher nicht zu / rather disagree
☐ weder noch / neither
☐ stimme eher zu / tend to agree
☐ stimme voll und ganz zu / fully agree

Web-Version: / web version:
☐ Stimme gar nicht zu / totally disagree
☐ Stimme eher nicht zu / rather disagree
☐ weder noch / neither
☐ stimme eher zu / tend to agree
☐ stimme voll und ganz zu / fully agree

6. Die Anwendung hat eine hohe Leistung. Das bedeutet: Die Anwendung hat kurze Ladezeiten. Es sind keine Fehler in der Anwendung vorhanden. / The application has a high performance. This means: The application has short loading times. There are no errors in the application.

App-Version: / app version:
☐ Stimme gar nicht zu / totally disagree
☐ Stimme eher nicht zu / rather disagree
☐ weder noch / neither
☐ stimme eher zu / tend to agree
☐ stimme voll und ganz zu / fully agree

Web-Version: / web version:
☐ Stimme gar nicht zu / totally disagree
☐ Stimme eher nicht zu / rather disagree
☐ weder noch / neither
☐ stimme eher zu / tend to agree
☐ stimme voll und ganz zu / fully agree

7. Die Anwendung ist benutzer:innenfreundlich. Das bedeutet: Die Anwendung ist intuitiv zu benutzen. Ich muss die Benutzung der Anwendung nicht lernen. Beschriftungen, Symbole und Anweisungen sind klar zu verstehen. / The application is user-friendly. This means that the application is intuitive to use. I don't have to learn how to use the application. Labels, symbols and instructions are clear to understand.

App-Version: / app version:
☐ Stimme gar nicht zu / totally disagree
☐ Stimme eher nicht zu / rather disagree
☐ weder noch / neither
☐ stimme eher zu / tend to agree
☐ stimme voll und ganz zu / fully agree

Web-Version: / web version:
☐ Stimme gar nicht zu / totally disagree
☐ Stimme eher nicht zu / rather disagree
☐ weder noch / neither
☐ stimme eher zu / tend to agree
☐ stimme voll und ganz zu / fully agree

8. Die Navigation in der Anwendung ist logisch und intuitiv. Das bedeutet: Ich kann einfach zwischen Bereichen wechseln. Es sind ausreichend Verlinkungen zwischen den Bereichen vorhanden. Das Wechseln ist logisch und nachvollziehbar. / Navigation in the application is logical and intuitive. This means: I can easily switch between areas. There are sufficient links between the areas. Switching is logical and comprehensible.

App-Version: / app version:
☐ Stimme gar nicht zu / totally disagree
☐ Stimme eher nicht zu / rather disagree
☐ weder noch / neither
☐ stimme eher zu / tend to agree
☐ stimme voll und ganz zu / fully agree

Web-Version: / web version:
☐ Stimme gar nicht zu / totally disagree
☐ Stimme eher nicht zu / rather disagree
☐ weder noch / neither
☐ stimme eher zu / tend to agree
☐ stimme voll und ganz zu / fully agree

9. Die Anwendung hat eine logische gestische Gestaltung. Das bedeutet: Das Bedienen der Anwendung ist logisch und intuitiv. Ich weiß immer, wann und wie getippt, geswiped und gescrollt werden muss. / The application has a logical gestural design. This means that operating the application is logical and intuitive. I always know when and how to type, swipe and scroll.

App-Version: / app version:
☐ Stimme gar nicht zu / totally disagree
☐ Stimme eher nicht zu / rather disagree
☐ weder noch / neither
☐ stimme eher zu / tend to agree
☐ stimme voll und ganz zu / fully agree

Web-Version: / web version:
☐ Stimme gar nicht zu / totally disagree
☐ Stimme eher nicht zu / rather disagree
☐ weder noch / neither
☐ stimme eher zu / tend to agree
☐ stimme voll und ganz zu / fully agree

10. Das Layout der Anwendung ist ansprechend. Das bedeutet: Die Gestaltung und Größe von Symbolen, Buttons, des Menüs und der Inhalte der Anwendung ist richtig und passend dargestellt. Ich kann alle Inhalte oder Funktionen erreichen. Es werden keine Inhalte oder Funktionen durch andere Sachen verdeckt. / The layout of the application is appealing. This means: The design and size of icons, buttons, the menu and the contents of the application are correctly and appropriately displayed. I can reach all content or functions. No content or functions are obscured by other things.

App-Version: / app version:
☐ Stimme gar nicht zu / totally disagree
☐ Stimme eher nicht zu / rather disagree
☐ weder noch / neither
☐ stimme eher zu / tend to agree
☐ stimme voll und ganz zu / fully agree

Web-Version: / web version:
☐ Stimme gar nicht zu / totally disagree
☐ Stimme eher nicht zu / rather disagree
☐ weder noch / neither
☐ stimme eher zu / tend to agree
☐ stimme voll und ganz zu / fully agree

11. Die Anwendung ist graphisch sehr gut aufbereitet. Das bedeutet: Die Grafiken, die für Buttons, Symbole, das Menü und die Inhalte verwendet werden, haben eine hohe Qualität und ein einheitliches Design. / The application has a very good graphic design. This means that the graphics used for buttons, icons, the menu and the content are of a high quality and have a standardized design.

App-Version: / app version:
☐ Stimme gar nicht zu / totally disagree
☐ Stimme eher nicht zu / rather disagree
☐ weder noch / neither
☐ stimme eher zu / tend to agree
☐ stimme voll und ganz zu / fully agree

Web-Version: / web version:
☐ Stimme gar nicht zu / totally disagree
☐ Stimme eher nicht zu / rather disagree
☐ weder noch / neither
☐ stimme eher zu / tend to agree
☐ stimme voll und ganz zu / fully agree

12. Die Anwendung sieht schön aus. Das bedeutet: Sie verwendet ansprechende Farben. Sie hat ein schönes Design, das auffällt und einprägsam ist. / The application looks beautiful. This means that it uses appealing colours. It has a beautiful design that stands out and is memorable.

App-Version: / app version:
☐ Stimme gar nicht zu / totally disagree
☐ Stimme eher nicht zu / rather disagree
☐ weder noch / neither
☐ stimme eher zu / tend to agree
☐ stimme voll und ganz zu / fully agree

Web-Version: / web version:
☐ Stimme gar nicht zu / totally disagree
☐ Stimme eher nicht zu / rather disagree
☐ weder noch / neither
☐ stimme eher zu / tend to agree
☐ stimme voll und ganz zu / fully agree

Die folgenden Fragen beziehen sich auf die gesamte Anwendung Steps. / The following questions relate to the entire Steps application.

13. Ich würde die Anwendung anderen Therapeut:innen empfehlen, die davon profitieren könnten. / I would recommend the application to other therapists who could benefit from it

 Ich würde die Anwendung niemanden empfehlen (überhaupt nicht) / I would not recommend the application to anyone (not at all)

 Ich würde die Anwendung nur wenigen Personen empfehlen/ I would only recommend the application to a few people

 Ich würde die Anwendung einigen Personen empfehlen (vielleicht) / I would recommend the application to some people (maybe)

 Ich würde die Anwendung vielen Personen empfehlen/ I would recommend the application to many people

 Ich würde die Anwendung jedem empfehlen (auf jeden Fall) / I would recommend the application to anyone (in any case)

14. Ich würde die Anwendung auch nach Abschluss des Projekts für meine Therapien nutzen. / I would also use the application for my therapies after the project has been completed.

 Nein / No
 Ja, für einzelne Patient:innen. / Yes, for individual patients.
 Ja, für alle weiteren Therapien. / Yes, for all other therapies.

15. Ich würde für die Anwendung auch Geld bezahlen. / I would also pay money for the application.

 Ja / yes Nein / no

16. Wenn ja: Wie viel Geld würdest du für die Nutzung der Anwendung monatlich ausgeben? / If yes: How much money would you spend per month to use the application?

______ in Euro / ______ in euros

17. Wie viele Sterne würdest du der Anwendung insgesamt geben? / How many stars would you give the application overall?

App-Version: / app version:
★ Eine der schlechtesten Anwendungen, die ich je genutzt habe / One of the worst applications I have ever used
★★
★★★ Durchschnittlich / Average
★★★★
★★★★★ Eine der besten Anwendungen, die ich je genutzt habe / One of the best applications I have ever used

Web-Version: / web version:
★ Eine der schlechtesten Anwendungen, die ich je genutzt habe / One of the worst applications I have ever used
★★
★★★ Durchschnittlich / Average
★★★★
★★★★★ Eine der besten Anwendungen, die ich je genutzt habe / One of the best applications I have ever used

Formative Evaluation: Qualitatives Feedback zu *Steps*

Formative evaluation: Qualitative feedback on *Steps*

Als letztes möchten wir von Ihnen mehr darüber erfahren, was Ihnen an der App gut oder schlecht gefallen hat. Wir möchten auch erfahren, welche Änderungsideen/-wünsche Sie für *Steps* haben.

Finally, we would like to know more about what you liked or didn't like about the app. We would also like to know what change ideas/wishes you have for *Steps*.

1. Welche Funktionen der App hat Ihnen in Ihrer therapeutischen Arbeit nicht geholfen?
   Finally, we would like to know more about what you liked or didn't like about the app. We would also like to know what change ideas/wishes you have for *Steps*.

---

2. Welche Funktionen haben Ihnen bei deiner Arbeit gefehlt? / Which functions did you miss in your work?

---

3. Was gefällt Ihnen an *Steps* besonders gut? / What do you particularly like about *Steps*?

---

4. Wo sehen Sie bei “Steps” Verbesserungspotential? / Where do you see potential for improvement in *Steps*?

---

5. Möchten Sie uns noch etwas Anderes mitteilen? / Is there anything else you would like to tell us?

1.3 Study three: Psychotherapy in private practices

1.3.1 Psychotherapists – Baseline survey

**Soziodemografie / Sociodemographics**
Zunächst ein paar Fragen zu Ihrer Person. / First of all, a few questions about yourself.

Wie alt sind sie? ___________ Jahre

How old are you? ___________ Years

Berufserfahrung: ___________ Jahre

Professional experience: ___________ years

Berufsqualifikation/ Professional qualification

- (Kinder- und Jugend-) Psychotherapeut:in in Ausbildung/ (Child and adolescent) psychotherapist in training
- (Kinder- und Jugend-) Psychotherapeut:in/ (Child and adolescent) psychotherapist
- Fachärzt:in für (Kinder- und Jugend-)psychiatrie und Psychotherapie/ Specialist in (child and adolescent) psychiatry and psychotherapy
- Sonstiges: _______/ Other: _______

Welcher Therapieschule fühlen Sie sich zugehörig? / Which therapy approach do you identify with?

- (Kognitive) Verhaltenstherapie/ (Cognitive) behavioural therapy
- Tiefenpsychologisch fundierte Psychotherapie / Depth psychology-based psychotherapy
- Systemische Therapie/ Systemic therapy
- Psychoanalyse/ Psychoanalysis
- Sonstiges: _______/ Other: _______

Medienaffinität: Besitz und Nutzung digitaler Medien

Media affinity: Ownership and use of digital media

Als nächstes möchten wir von Ihnen erfahren, ob und wie Sie digitale Medien beruflich nutzen.

Next, we would like to find out whether and how you use digital media at work.

1. Besitzen Sie ... ein Smartphone? / Do you own ... a smartphone?
   ☐ Ja ☐ Nein / ☐ Yes ☐ No

2. Besitzen Sie ... einen Computer? / Do you own … a computer?
   ☐ Ja ☐ Nein / ☐ Yes ☐ No

3. Besitzen Sie ... einen Laptop? / Do you own ... a laptop?
   ☐ Ja ☐ Nein / ☐ Yes ☐ No

4. Besitzen Sie ... ein Tablet? / Do you own ... a tablet?
   ☐ Ja ☐ Nein / ☐ Yes ☐ No

5. In Ihrem Alltag: Wie viel Zeit am Tag nutzen Sie durchschnittlich Ihr Smartphone oder Computer/Laptop/Tablet? / in your everyday life: How much time a day do you use your smartphone or computer/laptop/tablet on average?
   ☐ 0-2 Stunden / hours
   ☐ 2-4 Stunden / hours
   ☐ 4-6 Stunden / hours
   ☐ 6-8 Stunden / hours
   ☐ mehr als 8 Stunden / more than 8 hours

6. Nutzen Sie digitale Medien während Ihrer Sitzungen? / Do you use digital media during your meetings?
   ☐ Ja ☐ Nein / ☐ Yes ☐ No

7. Wenn ja: Welche digitalen Medien nutzen Sie? / If yes: Which digital media do you use?
   ____

8. Nutzen Sie Apps für Ihre Behandlung? / Do you use apps for your treatment?
   ☐ Ja ☐ Nein / ☐ Yes ☐ No

9. Wenn ja: Welche Apps nutzen Sie? / If yes: Which apps do you use?
   ____

10. Wenn ja: Haben Sie schon einmal eine DiGA (Digitale Gesundheitsanwendung) verschrieben? / If yes: Have you ever prescribed a DiGA (digital health application)?
 Ja Nein / Yes No

11. Erfassen Sie während der Probatorik Angaben zur Mediennutzung von Kindern und Jugendlichen? / Do you collect data on the media use of children and young people during the trial?
 nie / never
 manchmal / sometimes
 häufig / frequently
 immer / always

Affinity for Technology Interaction (ATI) (Franke, Attig & Wessel, 2018)

Im Folgenden geht es um Ihre Interaktion mit technischen Systemen. Mit „technischen Systemen" sind sowohl Apps und andere Software-Anwendungen als auch komplette digitale Geräte (z.B. Handy, Computer, Fernseher, Auto-Navigation) gemeint. Bitte geben Sie den Grad Ihrer Zustimmung zu folgenden Aussagen an:

The following is about your interaction with technical systems. By "technical systems" we mean apps and other software applications as well as complete digital devices (e.g. mobile phones, computers, televisions, car navigation systems). Please indicate your level of agreement with the following statements:

		Stimmt gar nicht / Not true at all	Stimme weitgehend nicht zu / Largely disagree	Stimmt eher nicht / Rather not true	Stimmt eher / Rather true	Stimmt weitgehend / Largely true	Stimmt völlig / Completely true
1	Ich beschäftige mich gern genauer mit technischen Systemen. I like to take a closer look at technical systems.	☐	☐	☐	☐	☐	☐
2	Ich probiere gern die Funktionen neuer technischer Systeme aus. I like to try out the functions of new technical systems	☐	☐	☐	☐	☐	☐

3	In erster Linie beschäftige ich mich mit technischen Systemen, weil ich muss. First and foremost, I deal with technical systems because I have to.	☐	☐	☐	☐	☐	☐
4	Wenn ich ein neues technisches System vor mir habe, probiere ich es intensiv aus. When I have a new technical system in front of me, I try it out thoroughly.	☐	☐	☐	☐	☐	☐
5	Ich verbringe sehr gern Zeit mit dem Kennenlernen eines neuen technischen Systems. I really enjoy spending time familiarising myself with a new technical system.	☐	☐	☐	☐	☐	☐
6	Es genügt mir, dass ein technisches System funktioniert, mir ist es egal, wie oder warum. It's enough for me that a technical system works, I don't care how or why.	☐	☐	☐	☐	☐	☐
7	Ich versuche zu verstehen, wie ein technisches System genau funktioniert. I try to understand exactly how a technical system works.	☐	☐	☐	☐	☐	☐
8	Es genügt mir, die Grundfunktionen eines technischen Systems zu kennen. It is enough for me to know the basic functions of a technical system.	☐	☐	☐	☐	☐	☐
9	Ich versuche, die Möglichkeiten eines technischen Systems vollständig auszunutzen. I try to fully utilise the possibilities of a technical system.	☐	☐	☐	☐	☐	☐

Einstellung zur Nutzung Moderner Technologien / Attitudes towards the use of modern technologies

MTPS (Bagarić & Jokić-Begić, 2020)

Nun möchten wir von Ihnen wissen, wie Ihre Einstellung zu modernen Technologien ist. Der Begriff „moderne Technologien“ bezieht sich auf: die Verwendung von PCs, Smartphones oder anderen Geräten; die Verwendung von Video- und Audiomaterialien, Webseiten, E-Büchern, Anwendungen und Programmen (z. B. Skype, Social Media, Viber, WhatsApp) für die Kommunikation mit Klienten, als Teil der therapeutischen Technik in der Sitzung oder als Teil der Aufgabe des Klienten zwischen den Sitzungen. Bitte geben Sie an, wie sehr Sie den folgenden Aussagen zustimmen.

Now we would like to know what your attitude to modern technologies is. The term "modern technologies" refers to: the use of PCs, smartphones or other devices; the use of video and audio materials, websites, e-books, applications and programs (e.g. Skype, social media, Viber, WhatsApp) for communication with clients, as part of the therapeutic technique in session or as part of the client's task between sessions. Please indicate how strongly you agree with the following statements.

		Stimme gar nicht zu / Totally disagree	Stimme eher nicht zu / Rather disagree	Weder noch / Neither	Stimme eher zu / Tend to agree	Stimme voll zu / Fully agree
1	Moderne Technologien können den therapeutischen Prozess beschleunigen. Modern technologies can speed up the therapeutic process.	☐	☐	☐	☐	☐
2	Moderne Technologien können die Wirksamkeit der Psychotherapie erhöhen. Modern technologies can increase the effectiveness of psychotherapy.	☐	☐	☐	☐	☐
3	Moderne Technologien können zu Vorteilen für Klient:innen führen, die mit anderen Methoden nicht erreicht werden können. Modern technologies can lead to benefits for clients that cannot be achieved with other methods.	☐	☐	☐	☐	☐
4	Moderne Technologien können den therapeutischen Prozess bereichern. Modern technologies can enhance the therapeutic process.	☐	☐	☐	☐	☐
5	Der Einsatz von modernen Technologien für die Psychoedukation kann nützlich sein (z. B. E-Books, E-Broschüren). The use of modern technologies for psychoeducation can be useful (e.g. e-books, e-brochures).	☐	☐	☐	☐	☐

6	Audio- und Videomaterial kann für therapeutische Zwecke nützlich sein.<br>Audio and video material can be useful for therapeutic purposes.	☐	☐	☐	☐	☐
7	Therapeut:innen sollten flexibel sein, was den Einsatz von modernen Technologien in der Psychotherapie angeht.<br>Therapists should be flexible when it comes to the use of modern technologies in psychotherapy.	☐	☐	☐	☐	☐
8	Multimediale Inhalte können bei einigen Klient:innen nützlich sein.<br>Multimedia content can be useful for some clients.	☐	☐	☐	☐	☐
9	Therapeut:innen sollten bei der Verwendung von modernen Technologien in der Psychotherapie sehr vorsichtig sein.<br>Therapists should be very careful when using modern technologies in psychotherapy.	☐	☐	☐	☐	☐
10	Es sollte eine strengere Regulierung der über moderne Technologien verfügbaren Inhalte in Bezug auf Psychotherapie und Psychopathologie durchgesetzt werden.<br>A stricter regulation of the content available via modern technologies in relation to psychotherapy and psychopathology should be enforced.	☐	☐	☐	☐	☐
11	Ein:e Therapeut:in sollte sich weigern, mit einem:einer Klient:in über moderne Technologien (Facebook, Viber, etc.) in Verbindung zu treten.<br>A therapist should refuse to connect with a client via modern technologies (Facebook, Viber, etc.).	☐	☐	☐	☐	☐
12	In einigen Fällen können moderne Technologien den therapeutischen Prozess beeinträchtigen.<br>In some cases, modern technologies can impair the therapeutic process.	☐	☐	☐	☐	☐
13	Ein:e erfahrene:r Therapeut:in hat keinen Bedarf an modernen Technologien.<br>An experienced therapist has no need for modern technologies.	☐	☐	☐	☐	☐

1.3.2 Psychotherapists – Post-survey

Was ist Ihre Meinung zu „Steps – Mein Weg zum Ziel“? / What is your opinion of "Steps - My way to my goal"?

____

Wahrgenommene App-Qualität / Perceived app quality

angelehnt an uMARS: uMARS (Stoyanov et al. 2016)

In den letzten Monaten konnten Sie die therapieunterstützende, transdiagnostische App "Steps - Mein Weg zum Ziel" intensiv kennenlernen und nutzen. Wir möchten nun gerne Ihre Meinung zu dieser App hören. Während der Therapien hatten Sie die Möglichkeit sowohl mit der App-Version für Patient:innen zu arbeiten als auch mit der webbasierten Therapeut:innen-Version. Deshalb möchten wir von Ihnen gerne erfahren, wie Sie beide Anwendungsmöglichkeiten erlebt haben und wie Sie die Qualität beider Anwendungsversionen einschätzen. Überlegen Sie sich deshalb bitte für jede Aussage, welche Antwort auf die App-Version und welche Antwort auf die webbasierte Therapeut:innen-Version am besten passt. Wir sind an Ihrer ehrlichen Meinung interessiert. Es gibt kein richtig oder falsch.

Over the past few months, you have had the opportunity to familiarise yourself with and use the therapy-supporting, transdiagnostic app "Steps - My way to my goal". We would now like to hear your opinion on this app. During the therapy sessions, you had the opportunity to work with both the app version for patients and the web-based therapist version. We would therefore like to find out how you experienced both application options and how you rate the quality of both application versions. For each statement, please consider which answer best fits the app version and which answer best fits the web-based therapist version. We are interested in your honest opinion. There is no right or wrong.

1. **Es macht Spaß, die Anwendung zu benutzen. / It's fun to use the application.**

App-Version: / app version:
☐ Stimme gar nicht zu / totally disagree
☐ Stimme eher nicht zu / rather disagree
☐ weder noch / neither
☐ stimme eher zu / tend to agree
☐ stimme voll und ganz zu / fully agree

Web-Version: / web version:
☐ Stimme gar nicht zu / totally disagree
☐ Stimme eher nicht zu / rather disagree
☐ weder noch / neither
☐ stimme eher zu / tend to agree
☐ stimme voll und ganz zu / fully agree

Welche Funktionen oder Dinge der Anwendung sorgen dafür, dass es (keinen) Spaß macht die Anwendung zu verwenden? Zum Beispiel im Vergleich zu anderen ähnlichen Anwendungen? / What features or things about the application make it (not) fun to use? For example, in comparison to other similar applications?

App-Version: / app version:
____
____

Web-Version: / web version:
____
____

2. Die Anwendung ist interessant zu benutzen. / The application is interesting to use.

App-Version: / app version:
☐ Stimme gar nicht zu / totally disagree
☐ Stimme eher nicht zu / rather disagree
☐ weder noch / neither
☐ stimme eher zu / tend to agree
☐ stimme voll und ganz zu / fully agree

Web-Version: / web version:
☐ Stimme gar nicht zu / totally disagree
☐ Stimme eher nicht zu / rather disagree
☐ weder noch / neither
☐ stimme eher zu / tend to agree
☐ stimme voll und ganz zu / fully agree

Welche Funktionen oder Dinge der Anwendung sorgen dafür, dass Sie die App interessant bzw. nicht interessant finden? Zum Beispiel im Vergleich zu anderen ähnlichen Anwendungen? / What functions or things in the application make you find the app interesting or not interesting? For example, in comparison to other similar applications?

App-Version: / app version:
____
____

Web-Version: / web version:
____
____

3. Die Anwendung ist individuell anpassbar. Das bedeutet: Ich kann die App nach meinen eigenen Wünschen anpassen (z.B. Töne, Benachrichtigungen oder Inhalte). Die Anpassungen werden gespeichert. Sie müssen nicht bei jeder Nutzung erneut eingestellt werden. / The application is customisable. This means: I can customise the app according to my own wishes (e.g. sounds, notifications or content). The customisations are saved. They do not have to be reset each time they are used.

App-Version: / app version:
☐ Stimme gar nicht zu / totally disagree
☐ Stimme eher nicht zu / rather disagree
☐ weder noch / neither
☐ stimme eher zu / tend to agree
☐ stimme voll und ganz zu / fully agree

Web-Version: / web version:
☐ Stimme gar nicht zu / totally disagree
☐ Stimme eher nicht zu / rather disagree
☐ weder noch / neither
☐ stimme eher zu / tend to agree
☐ stimme voll und ganz zu / fully agree

4. Die Anwendung ist interaktiv. Das bedeutet zum Beispiel: Ich kann eigene Inhalte eingeben, die App fordert mich zu Eingaben auf (z.B. durch Erinnerungen), ich erhalte Feedback durch die App oder habe die Möglichkeit mich über die App mit anderen auszutauschen. / The application is interactive. This means, for example: I can enter my own content, the app prompts me to make entries (e.g. through reminders), I receive feedback through the app or have the opportunity to exchange ideas with others via the app.

App-Version: / app version:
- ☐ Stimme gar nicht zu / totally disagree
- ☐ Stimme eher nicht zu / rather disagree
- ☐ weder noch / neither
- ☐ stimme eher zu / tend to agree
- ☐ stimme voll und ganz zu / fully agree

Web-Version: / web version:
- ☐ Stimme gar nicht zu / totally disagree
- ☐ Stimme eher nicht zu / rather disagree
- ☐ weder noch / neither
- ☐ stimme eher zu / tend to agree
- ☐ stimme voll und ganz zu / fully agree

5.Die Anwendung ist zielgruppenspezifisch. Das bedeutet: Die App und ihre Inhalte sind ansprechend für Jugendliche gestaltet. Sie hat eine ansprechende Optik und Sprache. Das Design ist ansprechend. / The application is target group-specific. This means that the app and its content are designed to appeal to young people. It has an appealing look and language. The design is appealing.

App-Version: / app version:
- ☐ Stimme gar nicht zu / totally disagree
- ☐ Stimme eher nicht zu / rather disagree
- ☐ weder noch / neither
- ☐ stimme eher zu / tend to agree
- ☐ stimme voll und ganz zu / fully agree

Web-Version: / web version:
- ☐ Stimme gar nicht zu / totally disagree
- ☐ Stimme eher nicht zu / rather disagree
- ☐ weder noch / neither
- ☐ stimme eher zu / tend to agree
- ☐ stimme voll und ganz zu / fully agree

6. Die Anwendung hat eine hohe Leistung. Die App hat kurze Ladezeiten. Es sind keine Fehler in der App vorhanden. / The application has a high performance. The app has short loading times. There are no errors in the app.

App-Version: / app version:
- ☐ Stimme gar nicht zu / totally disagree
- ☐ Stimme eher nicht zu / rather disagree
- ☐ weder noch / neither
- ☐ stimme eher zu / tend to agree
- ☐ stimme voll und ganz zu / fully agree

Web-Version: / web version:
- ☐ Stimme gar nicht zu / totally disagree
- ☐ Stimme eher nicht zu / rather disagree
- ☐ weder noch / neither
- ☐ stimme eher zu / tend to agree
- ☐ stimme voll und ganz zu / fully agree

7. Die Anwendung ist benutzer:innenfreundlich. Das bedeutet: Die App ist intuitiv zu benutzen. Ich muss die Benutzung der App nicht lernen. Beschriftungen, Symbole und Anweisungen sind klar zu verstehen. / The application is user-friendly. This means that the app is intuitive to use. I don't have to learn how to use the app. Labels, symbols and instructions are clear to understand.

App-Version: / app version:
☐ Stimme gar nicht zu / totally disagree
☐ Stimme eher nicht zu / rather disagree
☐ weder noch / neither
☐ stimme eher zu / tend to agree
☐ stimme voll und ganz zu / fully agree

Web-Version: / web version:
☐ Stimme gar nicht zu / totally disagree
☐ Stimme eher nicht zu / rather disagree
☐ weder noch / neither
☐ stimme eher zu / tend to agree
☐ stimme voll und ganz zu / fully agree

8. Die Navigation in der Anwendung ist logisch und intuitiv. Das bedeutet: Ich kann einfach zwischen Bereichen wechseln. Es sind ausreichend Verlinkungen zwischen den Bereichen vorhanden. Das Wechseln ist logisch und nachvollziehbar. / Navigation in the application is logical and intuitive. This means: I can easily switch between areas. There are sufficient links between the areas. Switching is logical and comprehensible.

App-Version: / app version:
☐ Stimme gar nicht zu / totally disagree
☐ Stimme eher nicht zu / rather disagree
☐ weder noch / neither
☐ stimme eher zu / tend to agree
☐ stimme voll und ganz zu / fully agree

Web-Version: / web version:
☐ Stimme gar nicht zu / totally disagree
☐ Stimme eher nicht zu / rather disagree
☐ weder noch / neither
☐ stimme eher zu / tend to agree
☐ stimme voll und ganz zu / fully agree

9. Die Anwendung hat eine logische gestische Gestaltung. Das bedeutet: Das Bedienen der App ist logisch und intuitiv. Ich weiß immer, wann und wie getippt, geswiped und gescrollt werden muss. / The application has a logical gestural design. This means that using the app is logical and intuitive. I always know when and how to tap, swipe and scroll.

App-Version: / app version:
☐ Stimme gar nicht zu / totally disagree
☐ Stimme eher nicht zu / rather disagree
☐ weder noch / neither
☐ stimme eher zu / tend to agree
☐ stimme voll und ganz zu / fully agree

Web-Version: / web version:
☐ Stimme gar nicht zu / totally disagree
☐ Stimme eher nicht zu / rather disagree
☐ weder noch / neither
☐ stimme eher zu / tend to agree
☐ stimme voll und ganz zu / fully agree

10. Das Layout der Anwendung ist ansprechend. Das bedeutet: Die Gestaltung und Größe von Symbolen, Buttons, des Menüs und der Inhalte der App ist richtig und passend dargestellt. Ich kann alle Inhalte oder Funktionen erreichen. Es werden keine Inhalte o-der Funktionen durch andere Sachen verdeckt. / The layout of the application is appealing. This means that the design and size of icons, buttons, the menu and the contents of the app are correctly and appropriately displayed. I can access all content or functions. No content or functions are obscured by other things.

App-Version: / app version:
☐ Stimme gar nicht zu / totally disagree
☐ Stimme eher nicht zu / rather disagree
☐ weder noch / neither
☐ stimme eher zu / tend to agree
☐ stimme voll und ganz zu / fully agree

Web-Version: / web version:
☐ Stimme gar nicht zu / totally disagree
☐ Stimme eher nicht zu / rather disagree
☐ weder noch / neither
☐ stimme eher zu / tend to agree
☐ stimme voll und ganz zu / fully agree

11. Die Anwendung ist graphisch sehr gut aufbereitet. Das bedeutet: Die Grafiken, die für Buttons, Symbole, das Menü und die Inhalte verwendet werden, haben eine hohe Qualität und ein einheitliches Design. / The application has a very good graphic design. This means that the graphics used for buttons, icons, the menu and the content are of a high quality and have a standardised design.

App-Version: / app version:
☐ Stimme gar nicht zu / totally disagree
☐ Stimme eher nicht zu / rather disagree
☐ weder noch / neither
☐ stimme eher zu / tend to agree
☐ stimme voll und ganz zu / fully agree

Web-Version: / web version:
☐ Stimme gar nicht zu / totally disagree
☐ Stimme eher nicht zu / rather disagree
☐ weder noch / neither
☐ stimme eher zu / tend to agree
☐ stimme voll und ganz zu / fully agree

12. Die Anwendung sieht schön aus. Das bedeutet: Sie verwendet ansprechende Farben. Sie hat ein schönes Design, das auffällt und einprägsam ist. / The application looks beautiful. This means that it uses appealing colours. It has a beautiful design that stands out and is memorable.

App-Version: / app version:
☐ Stimme gar nicht zu / totally disagree
☐ Stimme eher nicht zu / rather disagree
☐ weder noch / neither
☐ stimme eher zu / tend to agree
☐ stimme voll und ganz zu / fully agree

Web-Version: / web version:
☐ Stimme gar nicht zu / totally disagree
☐ Stimme eher nicht zu / rather disagree
☐ weder noch / neither
☐ stimme eher zu / tend to agree
☐ stimme voll und ganz zu / fully agree

13. Wie viele Sterne würden Sie *Steps* insgesamt geben? / How many stars would you give *Steps* overall?

App-Version: / app version:

★ Eine der schlechtesten Anwendungen, die ich je genutzt habe / One of the worst applications I have ever used
★★
★★★ Durchschnittlich / Average
★★★★
★★★★★ Eine der besten Anwendungen, die ich je genutzt habe / One of the best applications I have ever used

Web-Version: / web version:

★ Eine der schlechtesten Anwendungen, die ich je genutzt habe / One of the worst applications I have ever used
★★
★★★ Durchschnittlich / Average
★★★★
★★★★★ Eine der besten Anwendungen, die ich je genutzt habe / One of the best applications I have ever used

13. Ich würde die Anwendung anderen Therapeut:innen empfehlen, die davon profitieren könnten. / I would recommend the application to other therapists who could benefit from it.

- ☐ Ich würde die Anwendung niemanden empfehlen (überhaupt nicht) / I would not recomend the application to anyone (not at all)
- ☐ Ich würde die Anwendung nur wenigen Personen empfehlen/ I would only recommend the application to a few people
- ☐ Ich würde die Anwendung einigen Personen empfehlen (vielleicht) / I would recommend the application to some people (maybe)
- ☐ Ich würde die Anwendung vielen Personen empfehlen/ I would recommend the application to many people
- ☐ Ich würde die Anwendung jedem empfehlen (auf jeden Fall) / I would recommend the application to anyone (in any case)

14. Ich würde die Anwendung auch nach Abschluss des Projekts für meine Therapien nutzen./ I would also use the application for my therapies after the project has been completed.

☐ Nein / no
☐ Ja, für einzelne Patient:innen. / Yes, for individual patients.
☐ Ja, für alle weiteren Therapien. / Yes, for all other therapies.

15. Ich würde für die Anwendung auch Geld bezahlen. /I would also pay money for the application.

☐ Ja / yes ☐ Nein / no

16. Wenn ja: Wie viel Geld würdest du für die Nutzung der Anwendung monatlich ausgeben? If yes: How much money would you spend per month to use the application?
☐ Ja, ich wäre bereit dafür monatlich _____ Euro zu bezahlen. / Yes, I would be prepared to pay _____ euros per month.
☐ Nein / no

Formative Evaluation: Qualitatives Feedback zu *Steps*

Formative evaluation: Qualitative feedback on *Steps*

Instruktion für Interviewer:in: Die nächsten Fragen sind wieder offene, eigene Fragen. Die Fragen sind im Leitfaden thematisch gegliedert. Du musst nicht zwingend alle Fragen wortwörtlich stellen oder die Reihenfolge der Fragen einhalten.

Instruction for Interviewer: The next questions are again open questions of your own. The questions are organized thematically in the guide. You do not necessarily have to ask all the questions word for word or follow the sequence of questions.

Positive Aspekte & Chancen von Steps / Positive aspects & opportunities of Steps

- Was gefällt Ihnen an Steps besonders gut? / What do you particularly like about Steps?
- Welche Funktionen der App haben Ihnen in Ihrer therapeutischen Arbeit geholfen? / Which functions of the app have helped you in your therapeutic work?
- Welche Funktionen schätzen Sie als besonders hilfreich für Ihre Patient:innen ein? Welche Funktionen haben Ihren Patient:innen aus Ihrer Perspektive am besten gefallen? / Which functions do you consider to be particularly helpful for your patients? Which functions did your patients like best from your perspective?

Negative Aspekte & Herausforderungen von Steps / Negative aspects & challenges of Steps

- Was ist bei Ihren Patient:innen gar nicht gut angekommen? Gab es z.B. bestimmte Funktionen der App, die Patient:innen nicht nutzen wollten? / What didn't go down well with your patients? For example, were there certain functions of the app that patients did not want to use?
- Welche Funktionen haben Ihnen nicht geholfen? Gab es Funktionen, die Sie in Ihrer Arbeit gar nicht genutzt haben? / Which functions did not help you? Were there any functions that you did not use at all in your work?
- Welche Funktionen haben Ihnen für die therapeutische Arbeit gefehlt? / Which functions did you miss for your therapeutic work?
- Was waren Herausforderungen in der Arbeit mit Patient:innen (bezogen auf die App insgesamt)? Wo hat die App Ihre Arbeit erleichtert? / What were the challenges in working with patients (in relation to the app as a whole)? Where has the app made your work easier?

Ideen für die Weiterentwicklung / Ideas for further development

- Hatten Sie das Gefühl, dass die App für alle Patient:innen gleich hilfreich war? Oder gab es bestimme Störungsbilder/Symptomatiken bei denen die App besonders hilfreich war/überhaupt nicht hilfreich war? / Did you have the feeling that the app was equally helpful for all patients? Or were there certain disorders/symptoms for which the app was particularly helpful/not helpful at all?
- Haben Sie die App über die gesamte Therapie hinweg eingesetzt oder nur in bestimmten Therapiephasen? Würden Sie sagen, dass man die App in allen Therapiesituationen gleich gut einsetzen kann? Wo sehen Sie Unterschiede/Herausforderungen/Chancen? / Did you use the app throughout the entire therapy or only in certain phases of the therapy? Would you say that the app can be used equally well in all therapy situations? Where do you see differences/challenges/opportunities?
- Würden Sie die App nach dem Projekt gern weiternutzen? / Would you like to continue using the app after the project?
  - Wenn ja: Warum? / If yes: Why?
  - Wenn nein: Wie müsste die App verändert werden, damit Sie Steps gern weiterverwenden würden? / If not: How would the app have to be changed so that you would like to continue using Steps?
- *Stelle diese Frage nur Therapeutis, die aktiv mit der App gearbeitet haben:* Wir haben in diesem Projekt auch einen ersten Versuch gestartet Algorithmus-unterstützte Analysen für die Früherkennung von Hinweisen auf Suizidalität und nichtsuizidalem selbstverletzendem Verhalten einzusetzen. Haben Sie Anmerkungen oder Verbesserungsvorschläge für die Analysen der Stimmungs-Checks und der automatischen Textanalyse? / *Only ask this question to therapists who have actively worked with the app:* In this project, we have also started a first attempt to use algorithm-supported analyses for the early detection of indications of suicidal and non-suicidal self-harming behaviour. Do you have any comments or suggestions for improving the analyses of the mood checks and the automatic text analysis?
- Wo sehen Sie bei Steps weiteres Verbesserungspotential? / Where do you see further potential for improvement at Steps?
- Die Idee mit Steps ist eine beständige Therapiebegleitung für Jugendliche zu schaffen. Jugendliche sollen in ihrer stationären Behandlung die App kennenlernen und lernen damit zu arbeiten. Nach Entlassung können die Jugendlichen die App selbstständig weiternutzen, um mögliche Wartezeiten auf eine ambulante Therapie zu überbrücken. Und auch bei ambulanten Therapeut:innen können Jugendliche die App weiternutzen und so nahtlos an die Behandlung in der stationären Therapie und der Nachsorge anknüpfen. / The idea behind Steps is to create a permanent therapy support programme for young people. Young people should familiarise themselves with the app during their inpatient treatment and learn how to work with it. After discharge, the young people can continue to use the app independently in order to bridge any waiting times for outpatient therapy. Adolescents can also continue to use the app with outpatient therapists and thus seamlessly continue their treatment in inpatient therapy and aftercare.
- Was halten Sie von der Idee? Wo sehen Sie Potential/Herausforderungen? Was für Befürchtungen hätten Sie bei der Umsetzung einer App als beständiger Therapiebegleitung? Und wie müsste die App/das Netzwerk/das Projekt gestaltet sein, damit es realistisch umsetzbar wäre? / What do you think of the idea? Where do you see potential/challenges? What fears would you have about the implementation of an app as a permanent therapy

companion? And how would the app/network/project have to be designed for it to be realistically realisable?

- Zum Schluss möchten wir noch eine Frage ganz unabhängig von unserer Therapie-App Steps stellen: Wenn Sie sich die perfekte Therapie-App bestellen könnten: Wie sähe diese aus? / Finally, we would like to ask a question completely unrelated to our Steps therapy app: If you could order the perfect therapy app: What would it look like?
