# Supplementary material for: The Use of Mobile Apps in Adolescent Psychotherapy: Assessment of Psychotherapists’ Perspectives
Source: JMIR Form Res. 2025 Apr 8;9:e65788. doi: 10.2196/65788 (PMC12015344; doi:10.2196/65788)
Supplement: Multimedia Appendix 2 [file formative_v9i1e65788_app2.pdf]

Multimedia Appendix 2:

Final set of categories for the deductive qualitative content analysis

Categories that were added during the analysis are shown in italics.

APP-QUALITY (uMARS)		
Subject area	Code	Category name
Commitment	E01 (+ | -)	Possibility of customised design
	E02 (+ | -)	target group specific
	E03 (+ | -)	incentive/motivation to use the app (fun/interesting to use)
Functionality	F01 (+ | -)	ease of use (simplicity of using the app)
	F02 (+ | -)	performance (app performance, errors, error messages, loading times, functionality, ...)
Aesthetics	A01 (+ | -)	App graphic design
	A02 (+ | -)	Web graphic design
Information (=content/modules provided by the app)	*I01(+ | -)*	*Mood check*
	I02(+ | -)	*Goals & tasks*
	I03(+ | -)	*Logbook*
	I04(+ | -)	*Emergency kit*
	I05(+ | -)	*Providing therapy content/documentation*
	I14 (+|-)	*Behavioural analysis*
	I06A	*Library*
	I06	*Ideas for new/other functions*
	I07 (+ | -)	*Reminder function/appointments*
	I08 (+ | -)	*Avatar*
	I09 (+ | -)	*general aspects*
	I10 (+ | -)	*surveys*
	I11 (+ | -)	*algorithm-supported analyses of suicidality*
	I12 (+ | -)	*App as constant therapy support (inpatient - outpatient)*
	I13 (+|-)	*Communication options between patient & therapist*
Therapist-version	*I15 (+|-)*	*general aspects Therapeuti version*

FEASIBILITY		
inpatient specific aspects	*FE01 (+ | -)*	*changes in everyday ward life at the clinic*
	FE02 (+ | -)	*treatment smartphone (functions, use, ...)*
study specific aspects	*B12*	*study specific barriers (organisational effort, ...)*
	FE03 (+ | -)	*preparation phase (integration in project development, trainings, ...)*
	FE06 (+ | -)	*project monitoring (accessibility of the project leaders, study specific support possibilities, ...)*
general aspects	*FE04*	*extent of app use*
	FE05 (+ | -)	*other pros / cons of app use*
	FE08	*suitable for what kind of patients*
outpatient specific aspects	*FE07 (+ | -)*	*use in different status of therapy (diagnostic, therapeutic, in the end, ...)*

LEVEL OF ADOPTION OF E-MENTAL HEALTH MODEL		
Level	Code	Category name
GENERAL CHARACTERISTICS		
1 - 5	GC01 (+ | -)	Necessity of face to face contact
1 - 5	GC02 (+ | -)	Fitting to clients needs
BARRIERS		
1 - 5	B01	Technical Issues
1 - 3	B02	Conditions in daily practice: lack of time and resources, low visibility (workload, ...)
n.a.	*B02stat*	*Inpatient specific factors (only usable for psychotherapists, not for other professions, overwhelming for patients)*
n.a.	*B02set*	*Not fitting in the setting (recognition of outpatient use instead of inpatient treatment)*
1	B03	Feeling forced
1	B04	Unconvinced of benefits
1	B05	Dislike of mediated communication
1	B06	Lack of eHealth experience
2	B07	Sticking to automatic behaviour (integration in treatment context, own workflows)
2	B08	Limited knowledge of possibilites
3	B09	Feeling increased pressure of responsibility
3	B10	Limited knowledge of range of tools
4	B11	Small support base
n.a.	*B13*	*perceived risks of app use (data protection, harmful to the therapy process, ...)*
DRIVERS		
1 - 5	D01	Treatment quality: improvement of therapeutic alliance and accelerating therapy process
1 - 5	D02	Satisfaction of clients' needs
1 - 5	D03	Personal: efficiency, convenience, flexibility, access to documentation
2	D04	Belief in possible benefits
3	D05	Increasingly experiencing benefits
4 - 5	D06	Personal interest innovation eHealth
4 - 5	D07	New treatment possibilities
5	D08	Change of mental health care
n.a.	*D09*	*Support possibilities for the use of the app*
REQUIREMENTS FOR CHANGE		
2	RC01	Need of external trigger
	RC02	guiding principles
