# Supplementary material for: The Use of Mobile Apps in Adolescent Psychotherapy: Assessment of Psychotherapists’ Perspectives
Source: JMIR Form Res. 2025 Apr 8;9:e65788. doi: 10.2196/65788 (PMC12015344; doi:10.2196/65788)
Supplement: Multimedia Appendix 3 [file formative_v9i1e65788_app3.pdf]

Multimedia Appendix 3

3.1 Flow Chart of study participation presented for each study.

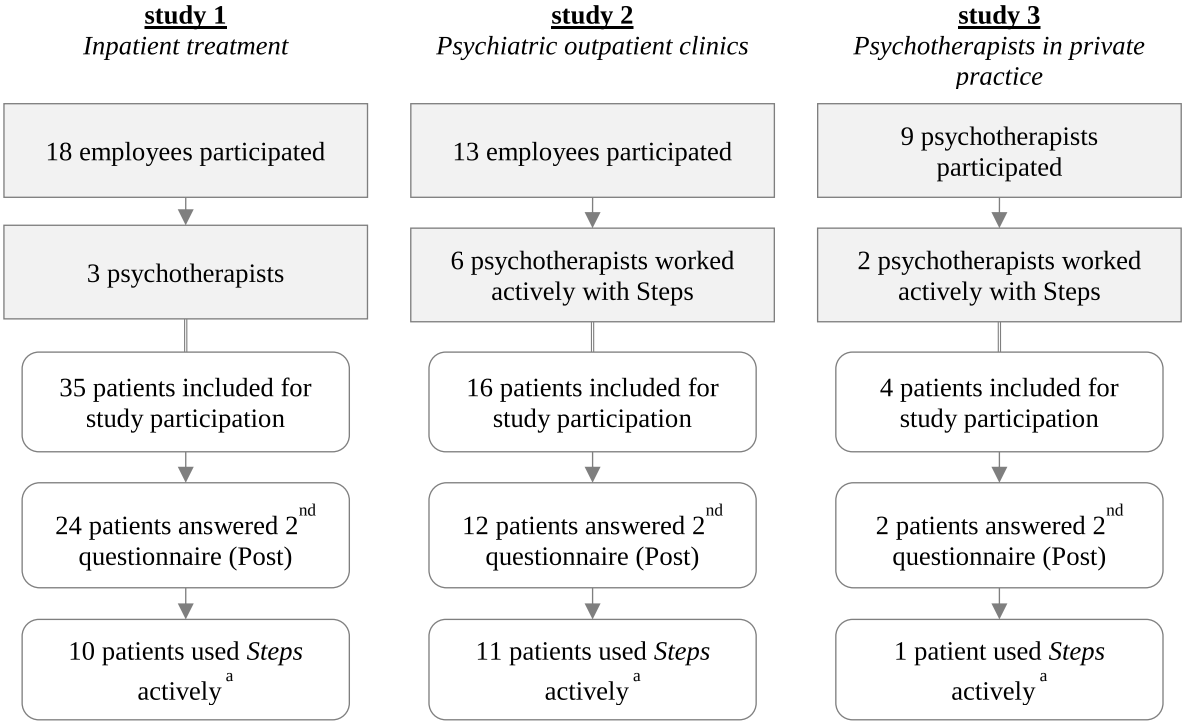

Notes. [a] active app use: mood checks were answered ≥ 10 times.

3.2 Frequencies of the self-created questions about the technical equipment available at work, the use of media and smartphone apps, and the integration of media in psychotherapies presented for studies two and three.

	Number of participants	
	Study two (n=13)	Study three (n=9)
Technical equipment available at work		
computer/laptop	13 (0 | 0) [a]	9 (0 | 0) [a]
Smartphone	3 (9 | 1) [a]	5 (4 | 0) [a]
tablet	2 (11 | 0) [a]	3 (4 | 2) [a]
Recommendation of mental health apps	3 (9 | 1) [a]	1 (8 | 0) [a]
Average daily media usage times		
0-2 hours	2	6
2-4 hours	4	0
4-6 hours	5	3
6-8 hours	2	0
> 8 hours	0	0

Notes. [a] numbers in brackets show the frequencies of equipment not available and missing answers.
